# High Proton Conductivity in *x*CuO/(1‐*x*)CeO_2_ Electrolytes Induced by CuO Self‐Nucleation and Electron‐Ion Coupling

**DOI:** 10.1002/advs.202417421

**Published:** 2025-03-27

**Authors:** Muhammad Shahid Sharif, Sajid Rauf, Zuhra Tayyab, Muhammad Ahsan Masood, Yibin Tian, Muhammad Ali Kamran Yousaf Shah, Abdullah N. Alodhayb, Rizwan Raza, Bin Zhu

**Affiliations:** ^1^ School of Energy and Environment Southeast University 2 Sipailou, Xuanwu District Nanjing 210096 China; ^2^ State Key Lab of Radio Frequency Heterogenous Integration & College of Mechatronics and Control Engineering Shenzhen University Shenzhen Guangdong 518060 China; ^3^ King Abdullah Institute for Nanotechnology King Saud University Riyadh 11451 Saudi Arabia; ^4^ Department of Physics COMSATS University Islamabad Lahore Campus Lahore 54000 Pakistan

**Keywords:** CuO/CeO_2_ heterostructures, CuO self‐nucleation, electron‐ion coupling, heterojunction engineering, semiconductor ionic membranes (SIMs)

## Abstract

Operating within the 300–500 °C range, low‐temperature solid oxide fuel cells (LT‐SOFCs) enable efficient and sustainable energy conversion, addressing the limitations of conventional high‐temperature SOFCs. However, achieving >0.1 S cm^−1^ ionic conductivity in electrolytes remains challenging. Here, a novel approach utilizing CuO self‐nucleation and electron‐ion (E‐I) coupling in *x*CuO/(1‐*x*) CeO_2_ (CCO) semiconductor ionic membranes (*x* = 0–0.4) is presented. At the optimal 0.2CuO/0.8CeO_2_ composition, ionic conductivity exceeds 0.15 S cm^−1^, driven by E‐I coupling at the CuO/CeO_2_ heterojunction. This coupling creates a built‐in electric field (BIEF) via interfacial charge transfer, facilitating ion transport by lowering the activation energy for ion migration. The dual‐conduction pathway enabled by E‐I coupling not only facilitates electronic transfer and ionic transport but also optimizes charge transfer kinetics, achieving exceptional power densities of 750–900 mW cm^−2^ at 500–550 °C and 78 mW cm^−2^ at 300 °C. Density functional theory (DFT) calculations further validate the role of Cu^2+^ and Ce^4+^ valence states in generating interfacial charge transfer and enhancing ionic mobility. This innovative approach positions CuO/CeO_2_ as a state‐of‐the‐art electrolyte, building the critical conductivity‐performance gap in LT‐SOFCs. This study pioneers LT‐SOFC innovation by leveraging E‐I coupling and electrode–electrolyte synergy, unlocking superior ion transport and practical applicability.

## Graphical abstract

This study discovers electron‐ion (E‐I) coupling in xCuO/(1‐x)CeO_2_(CCO), (x = 0–0.4) membranes, achieving ionic conductivity >0.15 S cm^−^^1^, with activation energy 0.28 eV and generated peak power density 900 mW cm^−^^2^ at 550 °C. Interfacial charge distribution and build‐in electric field enhanced ion transport entirely advanced the LT‐SOFC technology for clean energy conversion applications.

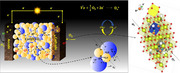

## 1 Introduction

Semiconductor ionic membranes (SIMs) represent a transformative approach in developing high‐performance LT‐SOFCs).^[^ ^1^ ^]^ Traditional SOFCs require high operating temperatures, typically above 800 °C, which limits material options, increases costs, and reduces system longevity.^[^ ^2^ ^]^ The drive for efficient energy conversion at low temperatures has motivated researchers to explore novel materials and mechanisms capable of surpassing conventional electrolyte limitations.^[^ ^3^ ^]^ One such innovation is the development of SIMs, which leverage mixed ionic‐electronic conduction to enhance fuel cell performance under the mild operational conditions.^[^ ^4^ ^]^

Mixed ionic‐electronic conductors (MIECs) are a frontier in SOFC technology, offering simultaneous ionic and electronic conduction that synergistically improves charge transport and electrochemical performance.^[^ ^1b^ ^]^ Unlike traditional electrolytes focused solely on ionic conduction, MIECs achieve dual conductivity, enhancing reaction kinetics and efficiency in fuel cells. SIM technology has enabled new configurations, such as single‐layer fuel cells (SLFCs) comprising homogeneous mixtures of electron‐ and ion‐conducting materials, to achieve performance comparable to traditional three‐layer designs.^[^ ^5^ ^]^ Balancing ionic and electronic conductivity is critical for optimizing electrochemical performance of FCs based on SIMs and MIECs.

Recent studies have underscored the potential of MIECs for LT‐SOFCs. For instance, Zhu et al. demonstrated that LiNi_0.1_Fe_0.9_O_2‐δ_ (LNF), a layered metal oxide, achieves both proton and electronic conduction in H_2_/air environments, reaching power densities of 640 and 760 mW cm^−2^ at 500 and 550 °C, respectively, when composited with samarium‐doped ceria (SDC).^[^ ^6^ ^]^ The composite formation between the semiconductor La_0.6_Sr_0.4_Co_0.2_Fe_0.8_O_3−δ_ (LSCF) and the as‐prepared Sm and Ca co‐doped ceria (SCDC), when integrated in an electrolyte‐layer‐free fuel cell (EFFC), achieved 814 mW cm^−2^ at 550 °C through oxygen vacancy management facilitated by enriched oxygen in the LSCF‐SCDC interface region.^[^ ^7^ ^]^ Additionally, a composite of p‐type semiconductor La_0.65_Sr_0.3_Ce_0.05_Cr_0.05_FeO_3‐δ_ (CLSCrF) and ionic conductor Ce_0.8_Sm_0.2_O_2‐δ_ (SDC) demonstrate a physical junction effect that prevented short circuits.^[^ ^8^ ^]^ Xing et al. further revealed that surface conduction in a CeO_2_/CeO_2‐δ_ core–shell structure can significantly enhance proton conductivity, reinforcing the potential of SIMs for LT‐SOFC.^[^ ^9^ ^]^

Considering previous bottle necks and following the literature, this study was investigated, where the *x*CuO/(1‐*x*)CeO_2_ (CCO) system was introduced as a new SIM material with electron‐ion (E‐I) coupling phenomenon to enhance ionic conduction for LT‐SOFCs. By varying the CuO content (*x* = 0‐0.4), we aim to optimize the balance between electronic transfer and ionic transport. In such way, the fine‐tuning of CCO composition can facilitate E‐I coupling, significantly enhancing ionic conductivity and overall fuel cell performance. By controlling sintering conditions, we promoted CuO self‐nucleation, where copper ions exhibit significant mobility within the ceria lattice, enriching grain boundaries and surfaces, resulting in the formation of *x*CuO/(1‐*x*)CeO_2_ heterojunction. The CuO self‐nucleation strategy in 0.2CuO/0.8CeO_2_ enables the formation of localized electronic transfer pathways, which synergistically enhance ionic conduction. Unlike conventional doping approaches that introduce aliovalent cations to create oxygen vacancies, CuO self‐nucleation forms a percolating network of Cu^2+^/Cu^+^ redox‐active sites that facilitate dynamic electron‐proton interactions. This mechanism not only stabilizes oxygen vacancies but also promotes defect‐assisted proton transport, effectively lowering activation energy and improving conductivity. Compared to previously reported doped ceria systems, which primarily rely on static oxygen vacancy formation for proton transport, the integrated E‐I coupling mechanism in our material enables ionic transport assisted by electronic transfer. This approach diverges from conventional electrolyte optimization by leveraging charge transfer to promote ionic transport. Experimental results show that *x*CuO/(1‐*x*)CeO_2_‐based SIMs achieve ionic conductivities exceeding 0.15 S cm^−1^. This enhancement is primarily attributed to E‐I coupling, where CuO phases in CCO enable the transition from semiconductor CeO_2_ to mixed electronic transfer assisted ionic conduction. This dual mechanism enhances ions transport and electro‐kinetics, yielding higher power density of the fuel cell device operation below 550 °C. The constructed fuel cell device based on optimized 0.2CuO/0.8CeO_2_ composition as electrolyte achieving 900 mW cm^−2^ at 550 °C, with minimized electrode polarization losses and improved overall cell performance. This approach offers a novel path for electrolyte optimization, driving a paradigm shift in low‐temperature ceramic fuel cell performance.

## 2 Results and Discussions

**Figure 1:**
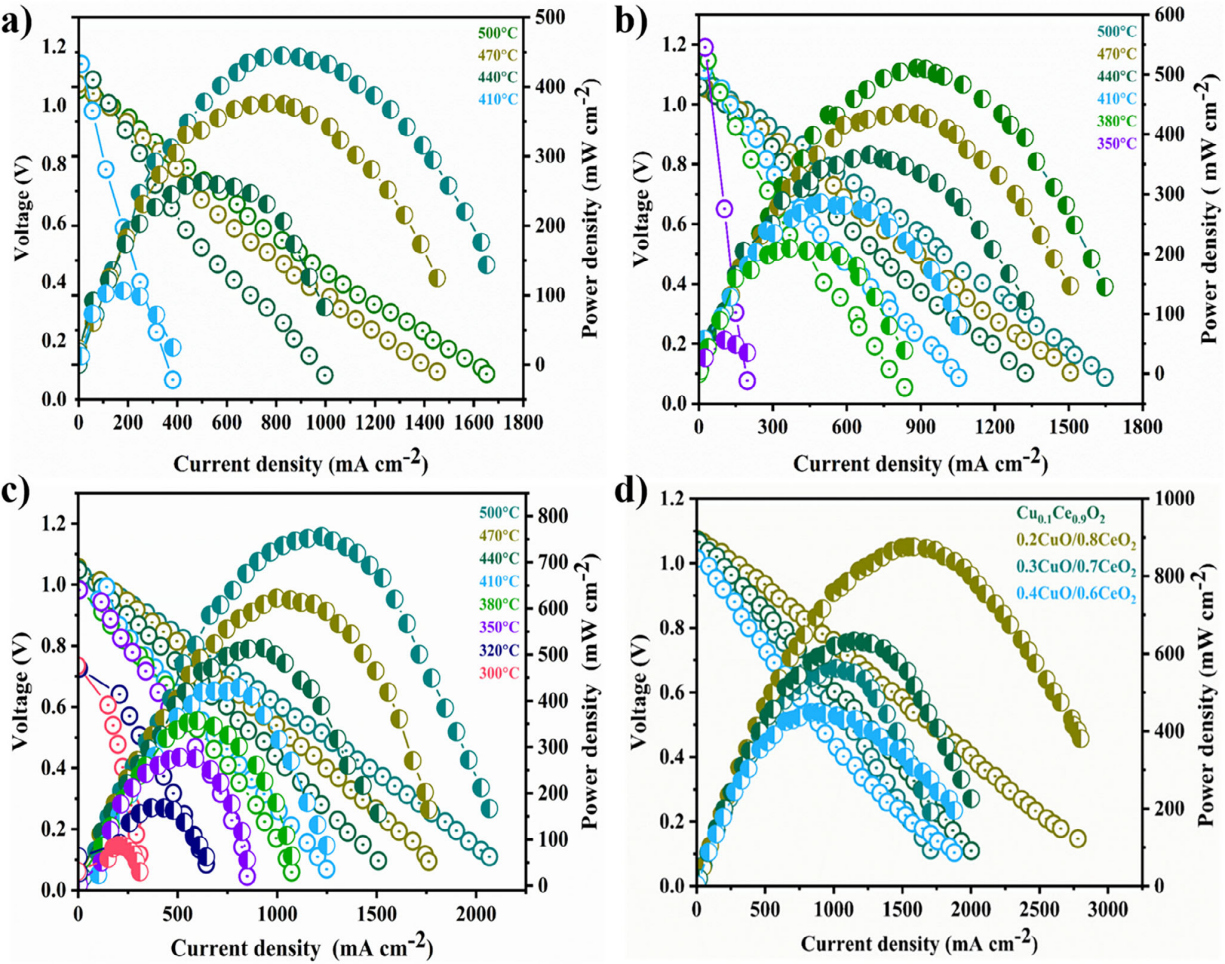
Fuel cell performance results in terms of *I–V* and *I–P* curves of various compositions *x*CuO/(1‐*x*)CeO_2_ (*x* = 0.1–0.4); a) CeO_2_. b) Cu_0.1_Ce_0.9_O_2_. c) 0.2CuO/0.8CeO_2_. d) Comparison between different compositions at 550 °C.

The fuel cell performance of *x*CuO/(1‐*x*)CeO_2_ (CCO)_compositions in **Figure** **1**a–d underscores the role of E‐I coupling in enhancing ionic conductivity and electrochemical efficiency, especially for low‐temperature solid oxide fuel cells (LT‐SOFCs). This coupling effect, facilitated by copper incorporation, establishes a synergy between charge transfer and ionic transport pathways, essential for achieving high power densities and operational stability across a wide temperature range. As shown in Figure 1a, pure CeO_2_ exhibits modest performance, with a steep decline in power density as temperature decreases due to its reliance solely on ionic conduction, lacking effective low‐temperature catalytic properties. On the other side, introducing Cu into CeO_2_ to synthesize Cu_0.1_Ce_0.9_O_2_ composition Figure 1b, significantly improved power density while spanning the operational temperature range to 350 °C as compared to pure ceria. Formation of a higher content of oxygen vacancy, the introduction of localized electronic states, and improved catalytic activity facilitate charge transfer is crucial for ion transport.^[^ ^10^ ^]^ This enables Cu_0.1_Ce_0.9_O_2_ to overcome the limitations of pure CeO_2_, allowing efficient operation at lower temperatures.

The 0.2CuO/0.8CeO_2_ composition Figure 1c achieves optimal E‐I coupling, delivering the highest power density and maintaining robust performance across temperatures. The balanced CuO/CeO_2_ heterostructure forms a built‐in electric field (BIEF) at the interface, reducing activation energy for ion migration and enhancing ionic transport (both are calculated and presented in the below sections). The mechanism of formation of localized electric fields and BIEF at interfaces facilitates faster charge transfer and reduces polarization losses, making 0.2CuO/0.8CeO_2_ electrolyte highly stable and efficient for LT‐SOFCs.^[^ ^11^ ^]^ The interface acts as a dynamic zone, where electronic transfer assisted ionic transport processes interact synergistically.^[^ ^12^ ^]^ Electrons from the CuO can easily migrate to the interface within the 0.2CuO/0.8CeO_2_ heterostructure, creating a surplus of electrons. This availability of electrons at the interface plays a crucial role in enhancing ion transport, leading to E‐I coupling, involving the transfer of electrons and ions creating a dynamic charge exchange process. However, Figure 1d and Figure S1a,b (Supporting Information) depict that increasing Cu content beyond 20% in 0.3CuO/0.7CeO_2_ and 0.4CuO/0.6CeO_2_ leads to a decline in the power output, as excessive electronic transfer overshadows ionic pathways. These findings illustrate the need for balanced E‐I contribution and coupling, where an optimal Cu concentration maximizes ionic conductivity and stability, marking 0.2CuO/0.8CeO_2_ as a promising electrolyte for LT‐SOFC applications.

### 2.1 Electrical Properties Investigation

**Figure 2:**
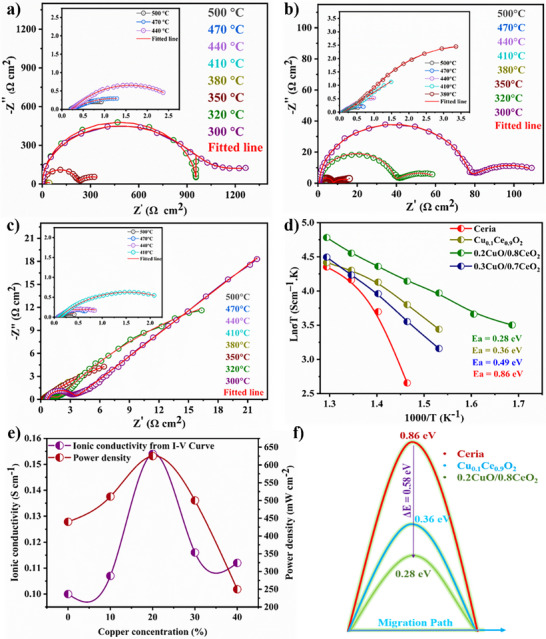
EIS simulated data of *x*CuO/(1‐*x*)CeO_2_ in various compositions: a) Ceria. b) Cu_0.1_Ce_0.9_O_2_. c) 0.2CuO/0.8CeO_2_. d) Activation energy plots of *x*CuO/(1‐*x*)CeO_2_ as a function of 1000/*T*. e) Plot representing changes in ionic conductivity and power density in response to increasing copper concentration. f) Decrease in potential barrier in terms of activation energies for ion migration.

The obtained Electrochemical Impedance Spectroscopy (EIS) raw data were fitted by designing an equivalent circuit (*R*_o_(*R*_1_‐QPE_1_)(*R*_2_‐QPE_2_), where the simulated EIS data are presented in **Figure** **2**a–c reveal strong evidence for the E‐I coupling effect in CCO compositions, particularly through the observed reductions in grain boundary resistance (*R*_gb_) and charge transfer resistance (*R*_ct_) with increasing Cu content. The high *R*_gb_ in pure CeO_2_ indicates significant ionic transport limitations, as grain boundaries (GB) create substantial barriers for ion migration. However, with heterojunction formation has noticeably decreased *R*_gb_, reaching its lowest values (0.034 Ω cm^2^) in 0.2CuO/0.8CeO_2_ at 500 °C. This reduction in *R*_gb_ suggests that charge transfer pathways introduced by CuO facilitate ionic transport across grain boundaries by minimizing energy barriers, thereby enhancing ionic conductivity through the E‐I coupling effect.^[^ ^13^ ^]^ Additionally, the decrease in *R*_ct_ across compositions highlights improved charge transfer efficiency at the electrode‐electrolyte interface. 0.2CuO/0.8CeO_2_ creates a BIEF at the CuO/CeO_2_ hetero‐interface, aligning oxygen ions and reducing activation energy for ion migration. This alignment promotes efficient charge transfer and accelerates electrochemical reactions, as evidenced by the low *R*_ct_ in 0.2CuO/0.8CeO_2_ heterojunction composition, further validating the E‐I coupling mechanism. A detailed comparison of *R*_b_, *R*_gb_, and *R*_ct_ values for all compositions is provided in Table S1 (Supporting Information).

The Arrhenius plot Figure 2d reinforces this mechanism, showing a decrease in activation energy (*E*a) from 0.86 eV for pure CeO_2_ to 0.28 eV for 0.2CuO/0.8CeO_2_, such a tremendous decrease of *E*a super increase the performance of the fuel cell device. Figure 2e illustrates the variation in ionic conductivity and power density with increasing copper concentration, highlighting the enhanced electrochemical performance of the optimized composition. Additionally, Figure 2f highlights the reduced potential barrier for ion transport, further supporting the role of copper incorporation in facilitating efficient ionic conduction. This decrease reflects the impact of E‐I coupling, as enhanced charge transfer lowers activation barriers, making ion migration easier. Beyond 20% Cu content, the excessive CuO promotes p‐type conductivity,^[^ ^10^ ^]^ overshadowing ionic pathways and increasing *E*a (0.49 eV at 30% CuO). This indicates that an optimal CuO concentration is critical in CCO for maximizing E‐I coupling benefits, and balancing electronic transfer and ionic transport for optimal LT‐SOFC performance. Figure S2a,b (Supporting Information) illustrates the EIS spectra for 0.3CuO/0.7CeO_2_ and 0.4CuO/0.6CeO_2_, Figure S2c (Supporting Information) displays DC polarization curves, demonstrating an increase in electronic transfer for *x*CuO/(1‐*x*)CeO_2_ (*x* = 0.1–0.3) with increased copper concentration.

To understand in detail, the charge transfer and ionic transport via BIEF and E‐I coupling acts as dual functionalities in facilitating ionic movement in the electrolyte membrane to enhance the electrode hydrogen oxidation reaction (HOR) and oxygen reduction reaction (ORR). The improved conductivity in CuO/CeO_2_ heterostructure as compared to CeO_2_ is attributed to reduced *E*a and enhanced ion‐coupling mechanisms.

We introduce ∆*E* represented as difference in *E*a between ionic and E‐I coupled ionic transport (Equation (1)), i.e.

(1) $$\Delta E\;\mathrm{for}\;\mathrm{electron}-\mathrm{ion}\;\mathrm{coupling}=Ea\left(\mathrm{ion}\right)-Ea\left(e-i\;\mathrm{coupled}\right)$$

And conductivity (Arrhenius equation) can be written as following Equation (2)

(2) $$\sigma =\sigma 0\;\exp\left(-Ea\;/\;\mathrm{kB}\;T\right)$$

Thus, E‐I coupled 0.2CuO/0.8CeO_2_ exhibited a 66% reduction in *E*a (0.28 eV compared to 0.86 eV for non E‐I coupling CeO_2_), resulting in also higher conductivity, 0.16 S cm^−1^ versus 0.1 S cm^−1^ for CeO_2_ at 773 K. The formation of CuO/CeO_2_ heterojunction and CuO presence at GB not only enhances charge transfer but also mobile ion concentration and modifies the local electrostatic environment, creating interfacial field at interface/GB.^[^ ^14^ ^]^ This interfacial field minimizes the energy barriers for ion hopping by aligning charge carriers more effectively with conduction pathways, enabling a shift from isolated, discrete transport to more continuous and delocalized motion. This transformation in ion transport characteristics is evident in the substantial increase in ionic conductivity (Figure 2e), signifying the role of CuO interfaces in facilitating cooperative ion migration.

Comparing the ionic conductivity of 0.2CuO/0.8CeO_2_ (0.15 S cm^−1^) at 500 °C with previous studies such as (CeO_2_ acted as electrolyte, which exhibits a considerable ionic conductivity of 0.1 S cm^−1^ at 550 °C,^[^ ^15^ ^]^ La_0.6_Sr_0.4_Co_0.2_Fe_0.8_O_3_ (calcined crab shell (CCS)‐LSCF) electrolyte exhibited proton conductivity of 0.115 S cm^−1^ was achieved at 550 °C,^[^ ^16^ ^]^ CeO_2_ exhibits a ionic conductivity of 0.13 S cm^−1^ at 550 °C,^[^ ^17^ ^]^ and SFT‐SDC heterostructure exhibits a high ionic conductivity 0.1 S cm^−1^ at 520 °C^[^ ^18^ ^]^ demonstrates that our material achieves superior conductivity at a relatively low operational temperature, showcasing its potential for enhanced performance in LT‐SOFC applications. In 0.2CuO/0.8CeO_2_, leveraging CuO self‐nucleation and E‐I coupling, achieves a lower *E*a (0.28 eV) and efficient operation from 300 to 500 °C. This dual‐conduction pathway enabled superior low‐temperature ionic conductivity and power density compared to conventional LT‐SOFC / SIMs, as summarized in **Table** **1**, positioning it as a strong candidate for next‐generation LT‐SOFCs.

**Table 1:** Performance comparison of 0.2CuO/0.8CeO_2_ with LT‐SOFC and SIM electrolytes, showcasing its optimized E‐I coupling for superior ionic transport and power output at low temperatures.

Electrolyte	Ionic conductivity [S cm^−1^]	Power density [mW cm^−2^]	Operational temperature [°C]	Lowest operational temperature [°C]	Refs.
Sr_0.7_Co_0.3_FeO_3−_ *_δ_*	0.13	771	550	450	[19]
Sm‐Pr co‐doped ceria	0.062	N‐A	750	500	[20]
LSCF‐CeO_2_	0.11	501	520	460	[21]
CeO_2_‐CoAl_2_O_4_	0.13	758	550	450	[22]
CeO_2‐δ_/CeO_2_	0.10	660	550	400	[15]
1LSCrF‐xCeO_2_	0.11	735	550	370	[23]
0.2CuO/0.8CeO_2_	0.15	750	500	300	This study

The detail is provided in order to perform the comparison of the current study based on SOFC with the proton exchange membrane fuel cells (PEMFCs). Actually, PEMFCs achieve high proton conductivity at 60–80 °C through hydrated membranes, their reliance on external humidification, precise humidity control, and high‐purity hydrogen significantly limits their long‐term stability and fuel flexibility. In contrast, the 0.2CuO/0.8CeO_2_ electrolyte demonstrates exceptional proton conductivity (0.15 S cm^−1^ at 500 °C) via vacancy‐mediated transport, ensuring sustained performance without the constraints of membrane hydration. The performance is facilitated by CuO self‐nucleation, which induces a dual‐conduction mechanism through E‐I coupling, resulting in *E*a of 0.28 eV, demonstrated significant ionic conductivity and power density within the 300–400 °C range focusing on (LT‐SOFC) applications. This low activation energy ensures efficient oxygen ion and proton migration at reduced temperatures, confirming the material's suitability for LT‐SOFCs. While peak power output (750 mW cm^−2^) is observed at 500 °C, the substantial conductivity and power output at 300–400 °C underscore the material's potential for efficient operation within the LT‐SOFC regime. Furthermore, the elevated operating temperature enhances system efficiency, enabling direct thermal integration in combined heat and power (CHP) setups, where overall efficiencies exceed 85%, due to waste heat recovery.^[^ ^24^ ^]^ Beyond conductivity, SOFCs offer superior fuel versatility, efficiently utilizing ammonia, biogas, and hydrocarbons through internal reforming, unlike PEMFCs, which require high‐purity hydrogen due to CO and sulfur sensitivity while also demonstrating greater durability by surface modifications and interface engineering, as oxide‐based electrolytes resist redox cycling and mechanical degradation, unlike polymer membranes prone to long‐term performance losses.^[^ ^24^, ^25^ ^]^

**Figure 3:**
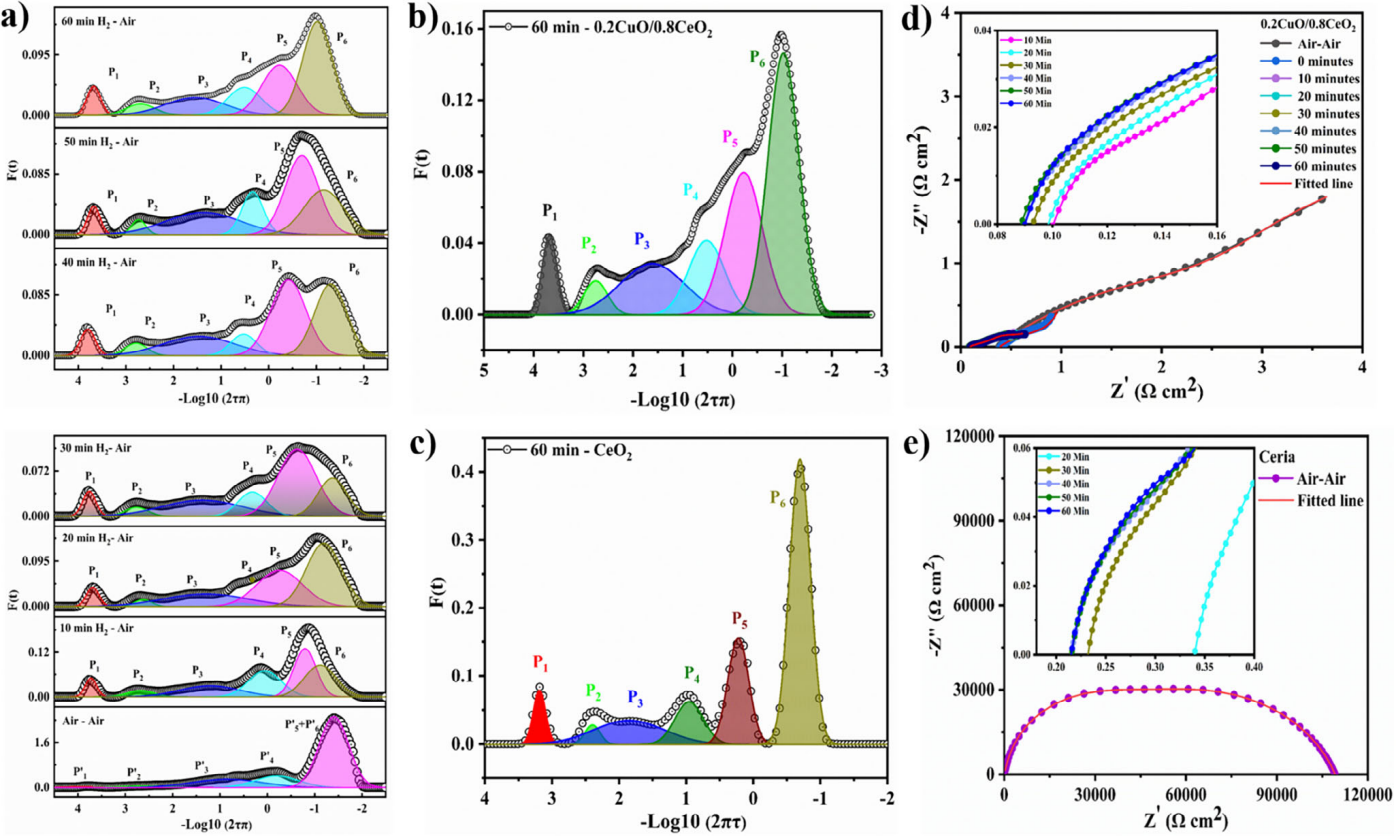
a) The DRT results for 0.2CuO/0.8CeO_2_ cell presenting sub‐processes involved to demonstrate the effect of electrochemical proton injection and grain boundary conduction from air/air to H_2_/air atmosphere till 60 min at 500 °C, b) DRT results for 0.2CuO/0.8CeO_2_ cell at 500 °C at 60 min of proton injection, c) DRT results for CeO_2_ cell at 500 °C at 60 min of proton injection. d) The Nyquist plot represents EIS data from air/air to H_2_/air atmosphere for 0.2CuO/0.8CeO_2_. e) for CeO_2_.

The distribution of relaxation time (DRT) profiles and EIS plots in **Figure** **3**a–e provide detailed insights into the E‐I coupling and synergistic H⁺/O^−2^ conduction mechanisms in the 0.2CuO/0.8CeO_2_ and CeO_2_ electrolytes. Figure 3a illustrates the evolution of DRT spectra for 0.2CuO/0.8CeO_2_ as it transitions from air/air to H_2_/air atmospheres over 60 min, highlighting dynamic changes in proton injection and interfacial/GB conduction. At 60 min, Figure 3b shows the resolved DRT peaks for 0.2CuO/0.8CeO_2_, while Figure 3c represents the DRT results for CeO_2_ under similar conditions. The CuO/CeO_2_ heterojunction significantly lowers polarization resistance across sub‐processes (P_1_‐P_6_) in 0.2CuO/0.8CeO_2_ compared to pure CeO_2_. For instance, P_1_, representing interfacial/GB conduction,^[^ ^26^ ^]^ shows a peak area reduction from 0.02031 (CeO_2_) to 0.01648 (0.2CuO/0.8CeO_2_), confirming enhanced proton transport through interface/GB (Tables S2 and S3, Supporting Information). This improvement is attributed to E‐I coupling, where CuO's charge transfer pathways facilitate proton migration, reducing impedance and energy barriers for protonic conduction. Additionally, Figure 3d,e compares the Nyquist plots for 0.2CuO/0.8CeO_2_ and CeO_2_, respectively, highlighting superior conductivity and reduced polarization resistance in 0.2CuO/0.8CeO_2_. The conductivity of 0.2CuO/0.8CeO_2_ rapidly increases under H_2_/air conditions, stabilizing at 0.556 S cm^−1^ after 60 min, compared to CeO_2_’s plateau of 0.256 S cm^−1^ (Figure S3b,c, Supporting Information). This enhancement is a direct result of E‐I coupling and the synergistic H^+^/O^2−^ conduction mechanism, where oxygen vacancies introduced at CuO/CeO_2_ heterojunction act as active sites for proton incorporation and transport. This promotes proton incorporation, diffusion, and improved ORR, as seen in Figure 3b,c and Figure S3 (Supporting Information). Over time, high‐frequency peaks decrease while low‐frequency peaks grow, reflecting ORR‐driven equilibria.^[^ ^26^, ^27^ ^]^ Ionic transport in solid oxide electrolytes reveals distinct mechanisms across frequency regions. In Figure 3b, the broad, collective response suggests delocalized, coupled ionic transport dynamics, which resulted in maximized ionic conductivity. Conversely, Figure 3c, characterized by sharp, localized peaks indicative of discrete ion hopping, corresponds to reduced ionic conductivity. Figure S4a,b (Supporting Information) further provides detailed electrochemical investigation for five layered based fuel cell with 0.2CuO/(0.8)CeO_2_ composition for protonic conduction evidence.

Furthermore, DRT analysis reveals peak shifts (P_1_‐P_4_) to higher frequencies for 0.2CuO/0.8CeO_2_ as compared to CeO_2_, indicating enhanced ionic conduction,^[^ ^28^ ^]^ due to coupled electronic transfer assisted ionic transport. For example, the high‐frequency peaks (P_1_ and P_2_), associated with surface proton injection and GB conduction,^[^ ^26^ ^]^ shift to higher frequencies in 0.2CuO/0.8CeO_2_, indicating faster ion dynamics (Tables S4 and S5, Supporting Information). Intermediate processes (P_3_ and P_4_), linked to proton bulk diffusion and ORR,^[^ ^26^, ^27^, ^28^ ^]^ also show reduced peak areas, confirming accelerated reaction kinetics in 0.2CuO/0.8CeO_2_. Notably, P_4_, corresponding to ORR, measured as 9463.42 Ω in CeO_2_, drops significantly in 0.2CuO/0.8CeO_2_ measured as 0.4828 Ω (Tables S2 and S3, Supporting Information) under air/air atmosphere. While just after 10 min of proton injection for both compositions, P_4_ drops to 0.1469 Ω (CeO_2_), and 0.06 Ω (0.2CuO/0.8CeO_2_), reflecting the shift from an O^2^⁻‐dominated pathway (O_2_ + 4e⁻ ↔ 2O^2^⁻) to a proton‐assisted pathway (2H⁺ + ½O_2_ + 2e⁻ ↔ H_2_O).^[^ ^29^ ^]^

Bode plot analysis further supports the synergistic effect between H^+^ and O^−2^ conduction (Figure S5a—h, Supporting Information). At high frequencies, 0.2CuO/0.8CeO_2_ shows lower phase angles than CeO_2_, indicating reduced capacitive effect due to enhanced charge transfer pathways.^[^ ^30^ ^]^ In mid‐frequency ranges, GB/interfacial ionic conduction in 0.2CuO/0.8CeO_2_ shows the strongest enhancement due to a smoother phase angle transition than pure ceria reflecting improved ionic transport facilitated by E‐I coupling.^[^ ^30^, ^31^ ^]^ Strong metallic interactions localized at metal‐oxide interfaces can stabilize regions with high defect density, creating favorable pathways for enhanced superionic conduction.^[^ ^14^, ^32^ ^]^ At lower frequencies, 0.2CuO/0.8CeO_2_ exhibits inductive behavior, absent in CeO_2_, confirming dynamic E‐I coupling, where transient electric fields guide ion migration. This behavior showcases CuO/CeO_2_’s charge transfer assisted ionic transport, facilitating efficient E‐I coupling and faster ORR/HOR kinetics.^[^ ^33^ ^]^ Subsequently, the combined effects of E‐I coupling and synergistic H⁺/O^2^⁻ conduction in 0.2CuO/0.8CeO_2_ lead to significant improvements in electrochemical performance, making it a superior candidate for LT‐SOFC applications. These findings underscore the importance of heterojunction engineering in achieving coupled electronic transfer‐assisted ionic transport.

### 2.2 Materials Characterizations

**Figure 4:**
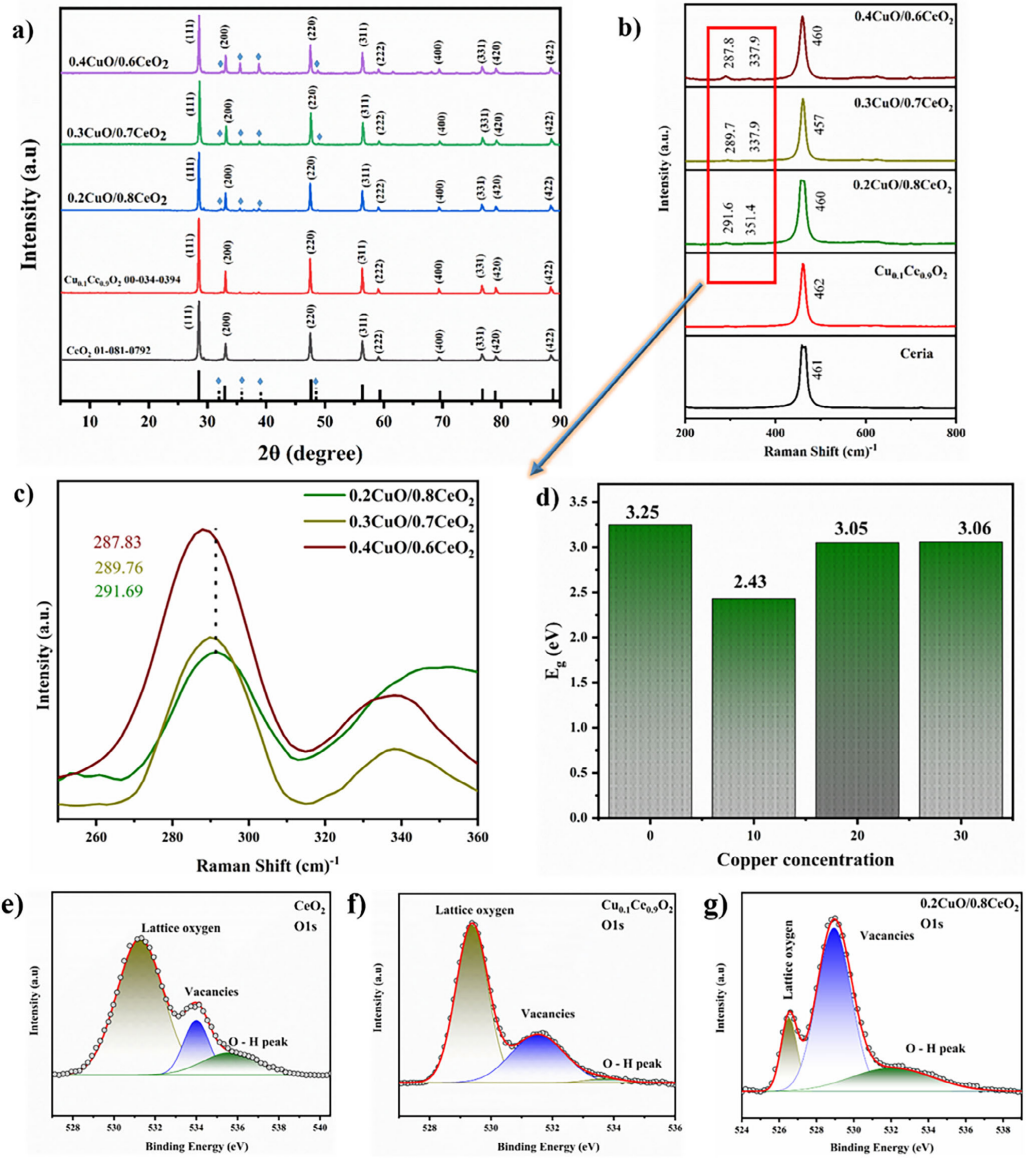
Physical characterization of prepared ceria and *x*CuO/(1‐*x*)CeO_2_ in various compositions, *x* = 0.1–0.4. a) XRD patterns. b,c) Raman analysis. d) Bandgap from the absorbance of UV–Vis graphs. e) O‐1s spectra analysis obtained for ceria. f) Cu_0.1_Ce_0.9_O_2_. g) 0.2CuO/(0.8)CeO_2_.

The material characterizations of the CCO composites, as presented in **Figure** **4**a–g and Figures S6–S8 (Supporting Information) offer insights into how CuO incorporation promotes E‐I coupling through structural, electronic, and morphological changes. The X‐ray diffraction (XRD) patterns in Figure 4a confirms the presence of CuO monoclinic structure (as the pristine monoclinic CuO phase shows diffraction peaks at 2*θ* = 32.41°, 35.44°, 38.73°, and 48.78°, corresponding to the (110), (−111), (111), and (202) planes, (JCPDS No. 89–2529)) and CeO_2_ fluorite structure across all *x*CuO/(1‐*x*)CeO_2_ compositions,^[^ ^34^ ^]^ with subtle lattice distortions indicating increased defect formation, particularly oxygen vacancies. No additional crystalline phases were observed, confirming the phase purity of the *x*CuO/(1‐*x*)CeO_2_ heterostructure. However, distinct diffraction peaks corresponding to the (−111) and (111) planes of monoclinic CuO were clearly identified in 0.2CuO/0.8CeO_2_, indicating the emergence of CuO alongside CeO_2_. This confirms the successful fabrication of CuO/CeO_2_ heterojunctions, highlighting the coexistence of both crystalline phases.^[^ ^35^ ^]^ Raman spectroscopy Figure 4b,c provides further confirmation as it shows shifts and additional vibrational features (especially at ≈289 cm^−1^_,_ related to the presence of CuO) in the CCO compositions,^[^ ^36^ ^]^ pointing to the formation of CuO/CeO_2_ heterostructure. In the 0.2CuO/0.8CeO_2_ heterostructure, CuO peaks are weak in XRD and Raman due to its low concentration, nanoscale CuO, and its surface coverage by CeO_2_ particles, which reduce CuO's peak intensity.^[^ ^36^, ^37^ ^]^ UV–Vis absorbance data Figure 4d and (Tauc plots added into Figure S7, Supporting Information, where the energy bandgaps were calculated and methodology is reported somewhere else^[^ ^38^ ^]^) reveal a narrowing bandgap (2.43 eV) with Cu‐doped CeO_2_ compared to CeO_2_ (3.25 eV), which stabilizes at ≈3.05 eV for CCO, indicating the formation of new electronic states that facilitate electron mobility. The narrowed bandgap implies a reduction in energy barriers, thus promoting charge transfer assisted ionic transport, which is essential for strong E‐I coupling in fuel cell applications. The X‐ray photoelectron spectroscopy (XPS) analysis of the O‐1s spectra Figure 4d–f further confirms an increase in oxygen vacancies with in 0.2CuO/0.8CeO_2_ composition. The O‐1s spectra show a decrease in the binding energy of lattice oxygen and oxygen vacancies, with 0.2CuO/0.8CeO_2_ exhibiting the lowest binding energy among the samples (Table S6, Supporting Information). Such an overall shift of peaks towards lower binding energy signifies an increase in the concentration of oxygen vacancies due to enhanced CuO content suggesting a more reactive oxygen environment that stabilizes charge carriers.^[^ ^39^ ^]^ The Scanning Electron Microscopy (SEM) images (Figure S8a,b, Supporting Information) confirm the formation of CuO/CeO_2_ heterostructure, showing well‐defined nanoscale grains and close‐packed particles which indicates strong interaction between CuO and CeO_2_ phases.

### 2.3 Structural Optimization and Investigation of Electronic Properties of Heterostructure

**Figure 5:**
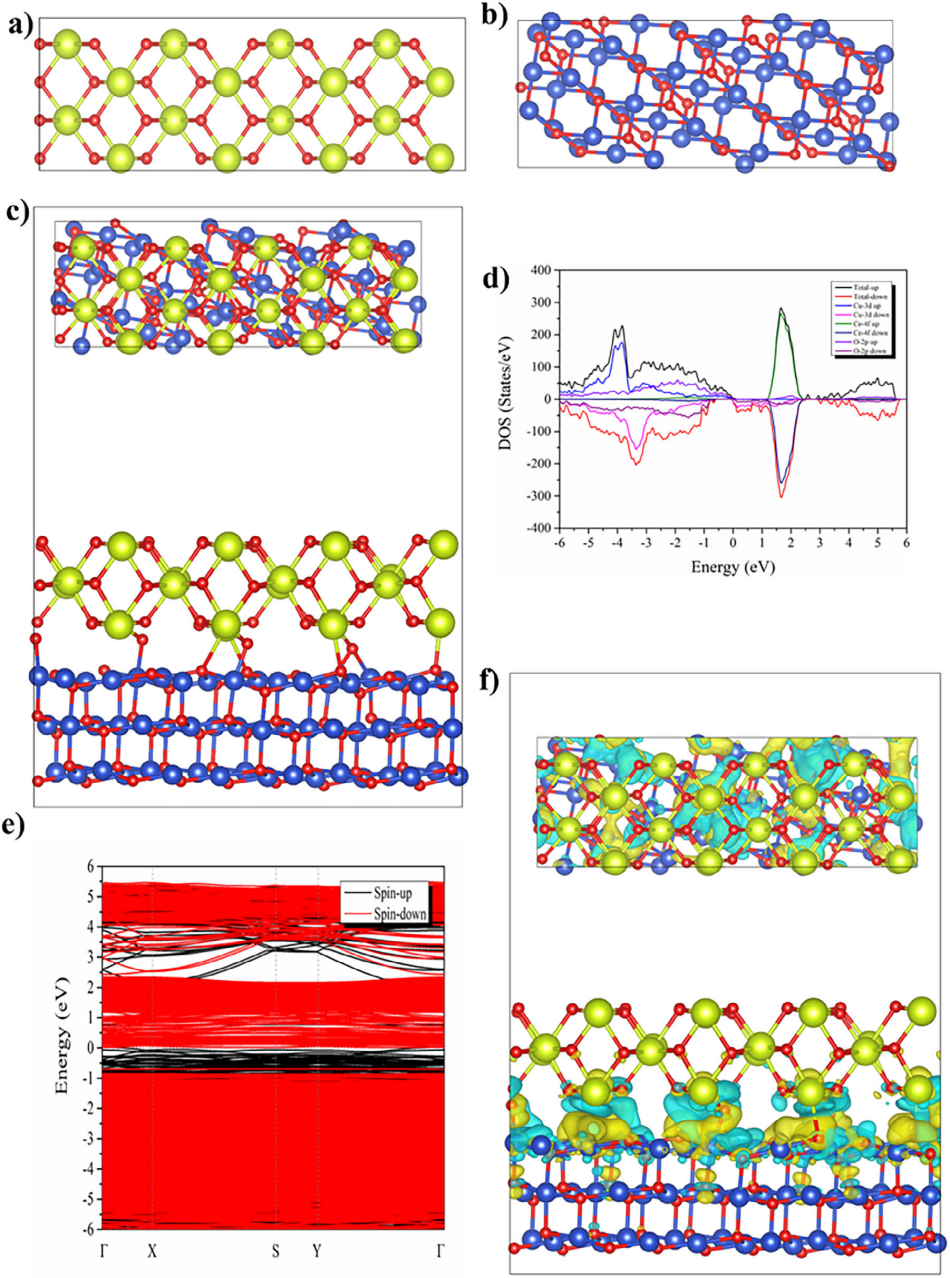
a) Optimized structure of ceria. b) CuO. (c) Side view of CuO–CeO_2_ heterostructure displayed the interface, inset shows top view of CeO_2_(110)/CuO (111) heterostructure interface. Ce, Cu, and O atoms are marked by yellow, blue, and red balls, respectively. d) Density of state of CuO/CeO_2_. e) Energy band structure of CuO/CeO_2_. f). Inset top and side view of charge density difference of CeO_2_/CuO heterostructure. Iso‐surface level was set at 0.01 e Å^−3^. Charge accumulation and depletion are indicated by yellow and cyan area, respectively. Charge transformation from CeO_2_ (110) to CuO (111) is 0.44879e. Ce, Cu, and O atoms are marked by yellow, blue, and red balls, respectively. The d‐band center was marked; where CuO was considered at (111), while CeO_2_ at (110).

The CeO_2_/CuO heterostructure depicted in **Figure** **5** offers a powerful platform for E‐I coupling, enabling synergistic transport mechanisms that enhance electrochemical performance in fuel cell and catalytic applications. The optimized structural models for CeO_2_ and CuO (Figure 5a,b) highlight the inherent properties of each component: CeO_2_ provides a stable lattice which supports oxygen ion conductivity, while CuO's layered structure facilitates electron mobility. These attributes are strategically aligned in the CeO_2_(110)/CuO(111) interface (Figure 5c), where CuO's conductive nature complements CeO_2_’s ionic conduction pathways. The CeO_2_/CuO interface fosters a unique environment by establishing a BIEF resulting from the disparity in electronic properties between CeO_2_ and CuO. This BIEF promotes charge transformation from CeO_2_ (110) to CuO (111) (calculated as 0.44879e). This charge redistribution creates local electric fields that influence both charge transfer and ionic conduction, enhancing redox reactions at the interface and facilitating proton migration across the structure, thereby achieving concurrent electronic transfer and ionic transport.^[^ ^40^ ^]^ This dual conductivity is essential for LT‐SOFCs, which rely on both electron and ion transport for efficient oxidation and reduction reactions.

Theoretical calculations further substantiate these observations. The density of states (DOS) and band structure analyses Figure 5d,e and (Figure S9a—d, Supporting Information) reveal the metallic nature of the CuO layer, characterized by bands crossing the Fermi level. The presence of Cu‐3d states near the Fermi level (in DOS) and the flat bands in the band structure confirm that CuO is the primary contributor to electronic conduction. CeO_2_ retains its semiconducting nature with a bandgap of 2.03 eV (Figure S9, Supporting Information), which supports oxygen vacancy formation, crucial for ionic transport. The combined presence of metallic and semiconducting behaviors at the interface enables strong E‐I coupling, allowing electronic charge from CuO to aid ionic movement in CeO_2_.

Charge density difference mapping Figure 5f indicates significant charge accumulation on the CuO side and depletion on the CeO_2_ side, with a net charge transfer of approximately 0.44879e from CeO_2_ to CuO. This charge redistribution at the atomic level generates local electric fields (LEF) that lower the migration barrier for ions, particularly for proton hopping between oxygen sites.^[^ ^41^ ^]^ The combination of charge redistribution and LEF facilitates proton transport across GB, as well as oxygen ion migration through oxygen vacancies, supporting dual charge transfer‐ionic transport pathways essential for fuel cell applications. Furthermore, Charge redistribution at the interface increases the average net charge of Cu atoms from 0.9639 (bulk CuO) to 1.0099 and slightly enhances the negative charge on oxygen atoms (‐1.0182), generating localized electric fields. In CeO_2_, charge disparity (Ce: +2.36, O: ‐1.17) facilitates proton transport via grain boundaries and oxygen vacancies by lowering the *E*a. This modified energy landscape enhances proton hopping, supported by complementary charge transfer, solidifying E‐I coupling as a key factor for SIMFC applications.

In consequence, the CeO_2_/CuO heterostructure design leverages E‐I coupling by combining CuO's charge transfer, with CeO_2_’s ionic transport pathways. This coupling is reinforced by the BIEF and charge redistribution at the interface, which enhances ion migration and redox processes, making the structure suitable for LT‐SOFCs and other energy applications requiring efficient E‐I coupling.

### 2.4 Coupled Electron Ion Conduction

**Figure 6:**
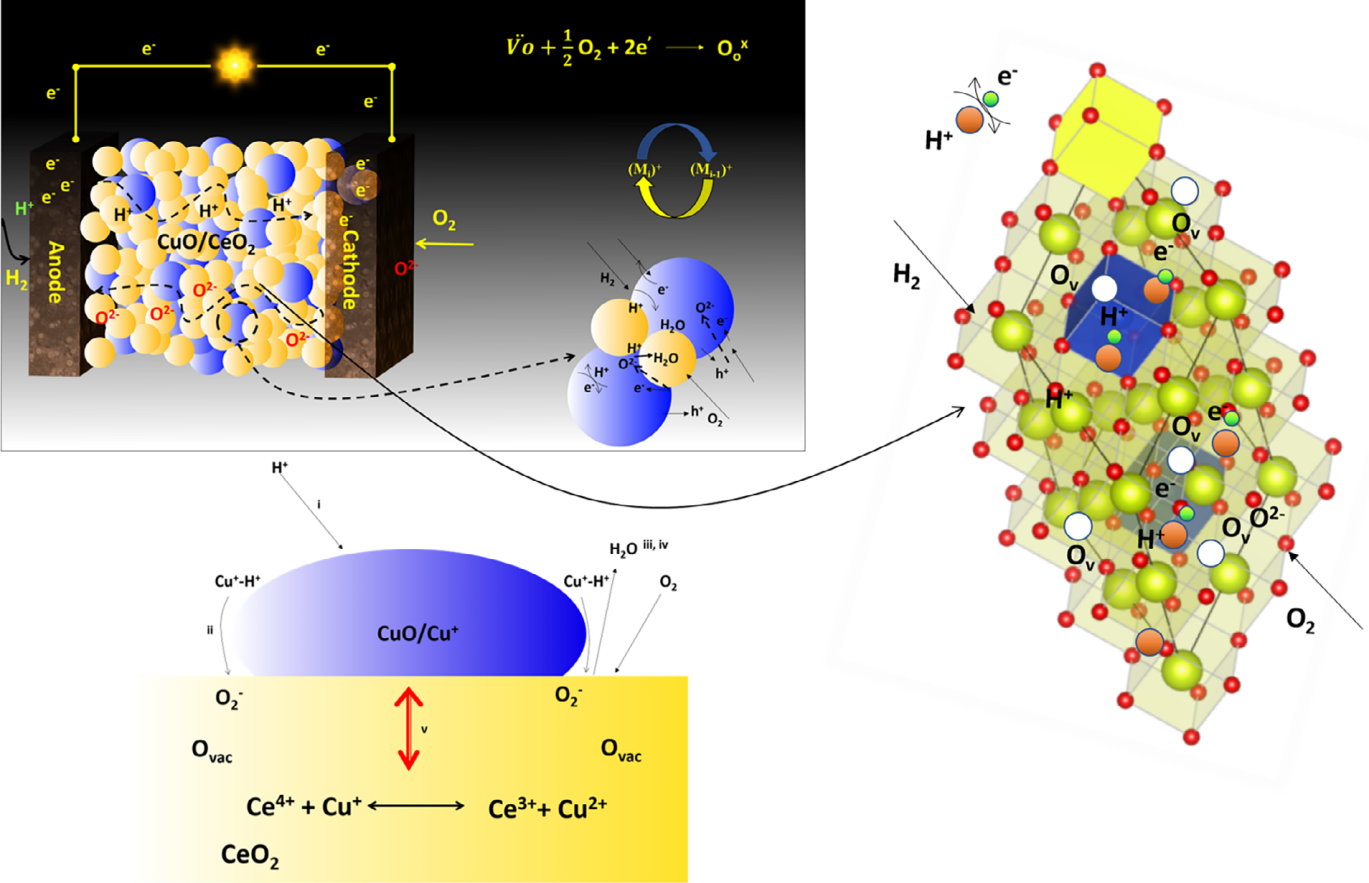
Schematic illustration for coupled electronic ionic conduction.

The schematic in **Figure** **6** illustrates the coupled E‐I conduction mechanism at the CuO/CeO_2_ heterostructure interface, highlighting critical steps that enable enhanced electrochemical performance in fuel cells. At this interface, interfacial charge redistribution (validated by DFT calculations) generates localized electrostatic fields that support E‐I coupling by guiding protons along CeO_2_ GB's.^[^ ^40^, ^41^, ^42^ ^]^ The presence of Ce^4+^ reduction to Ce^3+^ further facilitates oxygen vacancy formation, as confirmed by XPS analysis. Moreover, the interaction between CuO and CeO_2_ are postulated to be essential for Cu^+^ active species, as delineated by the redox equilibrium. This equilibrium is indicative of the redox interplay at interface that facilitates the formation of catalytically active sites within the material framework as reported in literature.^[^ ^43^ ^]^

This E‐I coupling mechanism operates through a sequential reaction process, i) H⁺ ions are chemisorbed onto Cu⁺ surface active sites, initiating the reaction. ii) These H⁺ ions then migrate toward the metal‐support interface, bringing them closer to CeO_2_, which contains accessible oxygen vacancies (O_x_). iii) Oxygen molecules from the air interact with oxygen vacancies on CeO_2_, creating active oxygen species that are primed for further reactions. iv) Chemisorbed H⁺ ions at the interface react with these active oxygen species, forming water (H_2_O) and facilitating charge transfer. v) Cu⁺ sites and oxygen vacancies are regenerated as gas‐phase oxygen replenishes the vacancies, allowing the reaction cycle to continue sustainably.

These steps underscore the continuous cycle that facilitates H_2_ and O_2_ reactions in the SOFC electrolyte, with E‐I conduction enhancing the overall redox activity. This coupling not only ensures efficient ion and electron transport but also maintains the redox balance within the structure, essential for catalytic performance and durability. Notably, the reduction of copper species in CuO/CeO_2_ at lower temperatures compared to pure CuO indicates a strong interaction between Cu and CeO_2_. During hydrogen reduction, Cu^2+^ is reduced to Cu⁺ at 773 K,^[^ ^43^, ^44^ ^]^ involving electron transfer (electronic conduction), while the reduction of Ce^4+^ to Ce^3+^ in CeO_2_ involves proton migration (ionic conduction). This simultaneous electron and ion transport is crucial for stabilizing intermediate oxidation states in copper, which may otherwise destabilize under similar conditions.

Thus, the synergy between electronic transfer (for copper reduction) and ionic transport (for ceria reduction) enhances both the reducibility and stability of the CuO/CeO_2_ system. The charge redistribution and LEF across the CuO/CeO_2_ interface optimize the material for LT‐SOFCs, where efficient coupled E‐I transport is necessary for high‐performance applications.

## 3 Conclusion

This study establishes xCuO/(1‐x)CeO_2_ (CCO) as a groundbreaking semiconductor ionic membrane (SIM) for low‐temperature solid oxide fuel cells (LT‐SOFCs), operating efficiently in the 300–500 °C range. The controlled sintering process at 750 °C induces CuO self‐nucleation forming the heterojunction with the CeO_2_ matrix, significantly enhancing electronion (E‐I) coupling, ionic conductivity, and oxygen vacancy generation. The optimal composition 0.2CuO/0.8CeO_2_ exhibits high ionic conductivity, exceeding 0.15 S cm^−1^, with decreased activation energies and enhanced fuel cell performance. Through distribution of relaxation times (DRT) analysis and density functional theory (DFT) calculations, this work reveals how interfacial E‐I coupling within the CuO/CeO_2_ heterostructure supports efficient charge transfer, enabling enhanced ionic transport promoted by electron coupling. This effect reduces also polarization losses across the electrolyte/electrode interface, advancing the state‐of‐the‐art in LT‐SOFCs by addressing traditional limitations in conductivity and electrolyte/electrode interface charge transfer efficiency at low temperatures. The findings highlight the significance of CCO membranes, offering a novel and transformative approach to SIMs in fuel cell applications. This work's novel insights into E‐I coupling set a new benchmark for fuel cell technology, positioning CCO as a viable pathway toward sustainable, high‐performance energy conversion. The tailored CCO SIM, with its scalable potential, marks a crucial advancement in LT‐SOFC technology and aligns with the global push for cleaner, low‐temperature energy solutions.

## 4 Experimental Section

### Synthesis of Proposed Materials

The *x*CuO/(1‐*x*)CeO_2_ (CCO) compositions (*x* = 0.1–0.4) were synthesized using the co‐precipitation method, leveraging nitrate precursors for their solubility in deionized (DI) water. Specific molar ratios for cerium (III) nitrate. nonahydrate (Sigma‐Aldrich, ACS 99% purity) and Copper nitrate hexahydrate (Sigma‐Aldrich, ACS 99%) were organized: 0.9:0.1, 0.8:0.2, 0.7:0.3, and 0.6:0.4. First, cerium nitrate was dissolved in of DI water (450 mL) and stirred at 70 °C, while copper nitrate was dissolved in DI water (120 mL) separately. Both solutions were mixed, stirred for 2.5 h, and sodium carbonate monohydrate (Sigma‐Aldrich, ACS 99% purity) solution (1_M_) was added slowly to induce precipitation (pH 7–8, adjusted with ammonia). The precipitate was filtered, washed with DI water and ethanol, dried at 180 °C for 12 h, then ground and sintered at 750 °C for 4 h. The resulting 0.2CuO/0.8CeO_2_ powder was used for subsequent analyses and fuel cell assembly. Other compositions followed the same method.

### Construction of Fuel Cell Device

The fuel cell structure included symmetrical Ni_0.8_Co_0.15_Al_0.05_LiO_2_ (NCAL) electrodes on either side of the 0.2CuO/0.8CeO_2_ electrolyte membrane. NCAL slurry was prepared using terpineol as the medium and applied to nickel foam then dried at 90 °C for 30 min. The electrolyte membrane was prepared by hydraulic pressing, and the cell assembly (Ni‐NCAL/0.2CuO/0.8CeO_2_ /NCAL‐Ni) was completed under 220 MPa, resulting in an active area of 0.64 cm^2^ and a cell thickness of 1.5 mm, with 520 µm electrolyte thickness. Nickel foam served as the current collector and provided mechanical stability. Similar procedures were applied for other compositions like Cu_0.1_Ce_0.9_O_2_, 0.3CuO/0.7CeO_2_, 0.4CuO/0.6CeO_2,_ and CeO_2_. Electrochemical performance was measured using an ITECH IT8511A+ DC electronic load under standard conditions, and electrochemical impedance spectroscopy (EIS) data were collected on a Solartron Energy Lab XM potentiostat over 0.1–10^5^ Hz at 10 mV AC in open‐circuit mode. For selective proton conduction assessment, a BaZr_0.8_Y_0.2_O_3_ (BZY)‐filtered five‐layer cell configured as (Ni‐NCAL/BZY/0.2CuO/0.8CeO_2_/BZY/NCAL‐Ni) was used. DC polarization studies using the Hebb‐Wagner method were conducted by activating the cell at 550 °C for 45 min, reducing to 500 °C, and subsequently switching from H_2_/air to nitrogen. Data were collected in a nitrogen atmosphere for 30 min. This process was repeated for all CCO compositions. Ionic and electronic conductivities were measured using EIS data, where total resistance (*R*_t_) was calculated as the sum of ohmic (*R*_0_) and grain boundary (*R*_1_) resistances. *I–V* and *I–P* curves were analyzed to determine ion transport efficiency.

EIS measurements were performed on a symmetrical NCAL/0.2CuO/0.8CeO_2_/NCAL cell, prepared via uniaxial pressing at 250 MPa to ensure uniform electrode contact. A Solartron 1260 impedance analyzer was employed, operating within a frequency range of 0.1 Hz to 1 MHz with an AC signal amplitude of 10 mV. To maintain stable experimental conditions, measurements were conducted in a controlled atmosphere, initially under air–air conditions, followed by proton injection at the anode side with repeated measurements taken at 10 min intervals. Hydrogen and air were supplied at a flow rate of 30 mL min^−1^ each, ensuring a consistent redox environment. For detailed impedance deconvolution, a distribution of relaxation times (DRT) analysis was conducted using an open‐source MATLAB‐based algorithm tailored for high‐resolution impedance spectra. The Tikhonov regularization approach was applied, with the optimal regularization parameter (*λ* = 10^−3^) determined via the L‐curve method to balance noise suppression and fitting accuracy. The selected frequency range (0.1 Hz to 1 MHz) allowed for capturing bulk, grain boundary, and interfacial relaxation processes. Characteristic peaks in the DRT spectrum were assigned to specific electrochemical phenomena based on their relaxation times, with assignments validated against literature benchmarks. To ensure consistency, peak fitting was cross‐verified across multiple datasets. Pre‐processing of impedance spectra was performed using Z‐Sim software to remove outliers, followed by MATLAB‐based computational analysis for DRT extraction and visualization. The analysis provided critical insights, including the identification of relaxation peak positions associated with grain boundary and mass transfer resistances, as well as a comparative evaluation of quantified resistances across different material compositions. The procedure was identically repeated for the CeO_2_‐based cell to ensure consistency in measurements and comparative analysis.

### Material Characterizations

Powder X‐ray diffraction (XRD) analysis was conducted on synthesized samples over a 2*θ* range of 10–90° using an analytical Empyrean diffractometer (Cu Kα radiation, 45 kV, 40 mA). Raman spectroscopy analyzed F2g band shifts related to the cubic fluorite structure. X‐ray photoelectron spectroscopy (XPS) assessed surface charge transfer, oxygen vacancies, and Ce^3+^/Ce^4+^ ratios using CASA XPS software. UV–Vis absorption spectroscopy (MIOSTECHPTY Ltd. UV3600) determined energy bandgaps. Scanning electron microscopy (SEM) examined surface morphology. Density functional theory (DFT) calculations were performed for structural optimizations via Vienna ab‐initio simulation package (VASP)^[^ ^45^ ^]^ with the projector augmented wave (PAW) method.^[^ ^46^ ^]^ The exchange‐functional was treated using the Perdew‐Burke‐Ernzerhof (PBE) functional, in combination with the DFT‐D3 correction.^[^ ^47^ ^]^ Cut‐off energy of the plane‐wave basis was set as 450 eV. For optimization of lattice size of slab models, the Brillouin Zone integration was performed with a Monkhorst‐Pack *k*‐point sampling of 1×3×1.^[^ ^48^ ^]^ The self‐consistent calculations applied a convergence energy threshold of 10^−5^ eV. The equilibrium geometries and lattice constants were optimized with maximum stress on each atom within 0.02 eV Å^−1^. The spin polarization method was used to describe magnetism of slab models. The Hubbard *U* correction method was introduced to describe Cu‐3d and Ce‐4f orbitals, where *U*_Cu_ = 4.0 eV, *U*_Ce_ = 5.4 eV.^[^ ^49^ ^]^ Band structure and density of states of slab models were calculated by GGA‐PBE functional, and then treated by VASPKit interface.^[^ ^50^ ^]^ Isosurface level of charge density difference was set at 0.01 e Å^−3^.

## Conflict of Interest

The authors declare no conflict of interest.

## Supporting information



Supporting Information

## Data Availability

The data that support the findings of this study are available from the corresponding author upon reasonable request.
