# Supplementary material for: High Proton Conductivity in xCuO/(1‐x)CeO2 Electrolytes Induced by CuO Self‐Nucleation and Electron‐Ion Coupling
Source: Adv Sci (Weinh). 2025 Mar 27;12(22):2417421. doi: 10.1002/advs.202417421 (PMC12165038; doi:10.1002/advs.202417421)
Supplement: Supplementary file 1 — Supporting Information [file ADVS-12-2417421-s001.docx]

**Supporting Data**

**High Proton Conductivity in xCuO/(1-x)CeO_2_ Electrolytes Induced by CuO Self-Nucleation and Electron-Ion Coupling**

*Muhammad Shahid Sharif^1^, Sajid Rauf^2,^*, Zuhra Tayyab^2^, Muhammad Ahsan Masood^1^, Yibin Tian^2,^*, M.A.K. Yousaf Shah^2^, Abdullah N.Alodhayb*^3^*, Rizwan Raza^4^, Bin Zhu^1,^**

^1^School of Energy and Environment, Southeast University, Nanjing, China.

^2^College of Mechatronics and Control Engineering, Shenzhen University, Shenzhen, Guangdong, China.

^3^Department of Physics and Astronomy, College of Science, King Saud University, Riyadh 11451, Saudi Arabia.

^4^Department of Physics, COMSATS University Islamabad, Lahore Campus, Pakistan.

**Correspondence to**

**Res. Assoc. Prof. Sajid Rauf:** [sajidrauf.physics@gmail.com](mailto:sajidrauf.physics@gmail.com) ; [sajidrauf@szu.edu.cn](mailto:sajidrauf@szu.edu.cn)

**Prof. Dr. Yibin Tian:** [ybtian@szu.edu.cn](mailto:ybtian@szu.edu.cn)

**Prof. Dr. Bin Zhu:** [zhu-bin@seu.edu.cn](mailto:zhu-bin@seu.edu.cn)

Figure S1a-b illustrates that raising the copper concentration resulted in current leakage below 450 °C for 0.3CuO/0.7CeO_2_, which can be more noticeable for 0.4CuO/0.6CeO_2_ composition. For 0.3CuO/0.7CeO_2_, it showed a lower OCV of 1.03V at 500 °C along with a decreased power output of 500 mW cm^-2^. On the other hand, its OCV and power density rapidly decrease at lower temperatures as evident from Figure S1a. Electrochemical analysis of a different as-prepared composition, identified as 0.4CuO/0.6CeO_2_, revealed only 225 mW cm^-2^ at 500 °C with an OCV of 0.7 V suggesting the occurrence of an ionic to electronic transition with rising copper concentration.


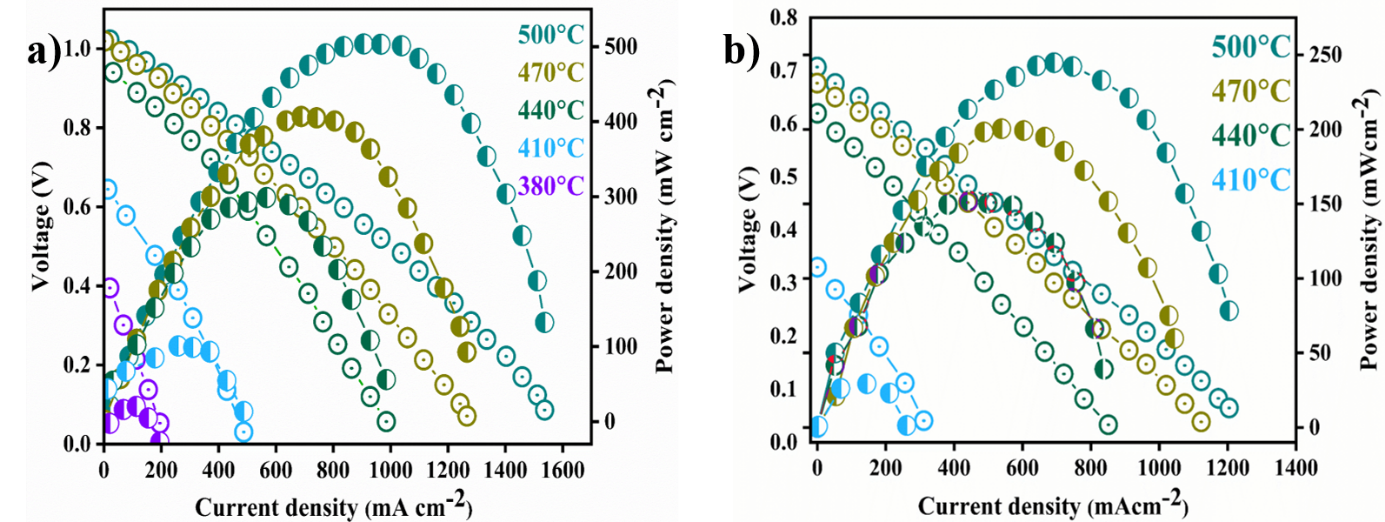


**Figure S1:** FC performance graphs of xCuO/(1-x)CeO_2_ compositions: (a) 0.3CuO/0.7CeO_2_ and (b) 0.4CuO/0.6CeO_2_.

Figure S2a-b shows Nyquist plot for 0.3CuO/0.7CeO_2_ and 0.4CuO/0.6CeO_2_ compositions, illustrating mixed ionic electronic conduction (MIEC) behavior. Hebb Wagner method of DC Polarization was considered to calculate electronic conductivity presented in Figure S2c. Prepared cell of Cu_0.1_Ce_0.9_O_2_ composition was tested under nitrogen atmosphere for 30 minutes approximately. At 500 °C, Cu_0.1_Ce_0.9_O_2_ composition provided 1.5 x 10^-2^ S cm^-1^ of electronic conductivity. Considering 0.2CuO/(0.8)CeO_2_, the electronic conductivity increased to 3.3 x 10^-2^ S cm^-1^ at 500 °C. While 0.3CuO/0.7CeO_2_ composition under same conditions provided higher increased electronic conductivity, calculated as 5.4 x 10^-2^ S cm^-1^. In the same manner, 0.4CuO/0.6CeO_2_ has electronic conductivity calculated as 6.4 x 10^-2^ S cm^-1^ as can be seen from Figure S2d.


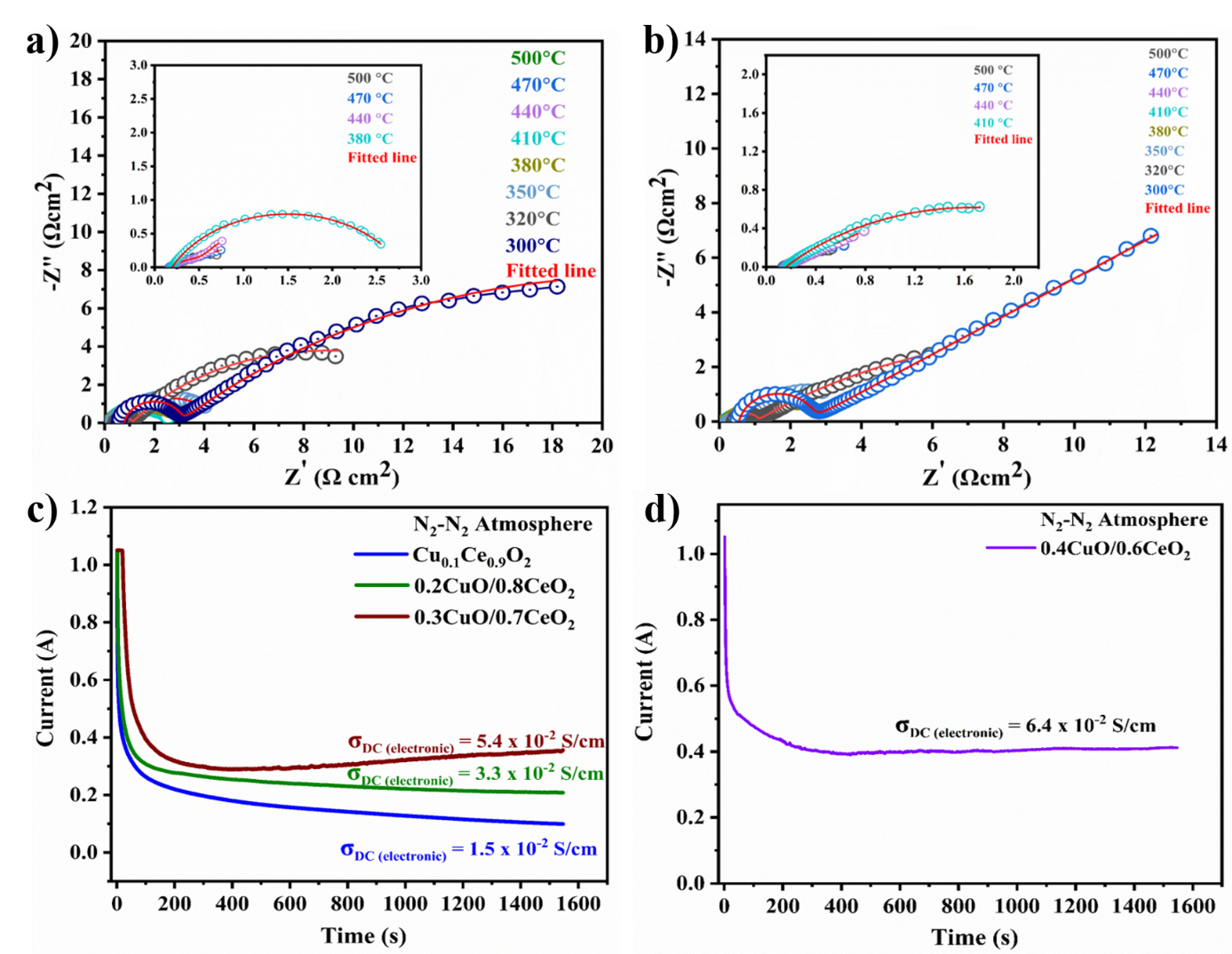


**Figure S2:** EIS of xCuO/(1-x)CeO_2_ compositions: (a) 0.3CuO/0.7CeO_2_, (b) 0.4CuO/0.6CeO_2_, (c) DC polarization curves illustrating the electronic conductivity behavior for xCuO/(1-x)CeO_2_ in various compositions, (d) for 0.4CuO/0.6CeO_2_ composition.

The DRT analysis supports a “two-stage model” of ionic conduction. First, rapid surface proton injection, is followed by slower bulk diffusion. This conductivity analysis reveals distinct behaviors between pure CeO_2_ and 0.2CuO/0.8CeO_2_ under H_2_-air conditions, with the latter demonstrating enhanced proton transport characteristics. While CeO_2_ shows a gradual increase in conductivity over time, indicative of limited proton injection and slower bulk transport, 0.2CuO/0.8CeO_2_ exhibits a rapid initial rise and achieves a significantly higher steady-state conductivity as evident from Figure S3b-c. This suggests that CuO heterojunction not only accelerates initial proton injection but also promotes sustained proton transport through increased oxygen vacancies, which serve as effective pathways for proton conduction. 0.2CuO/0.8CeO_2_ demonstrates significant improvements in proton transport and ORR performance. Enhanced DRT peaks, higher steady-state conductivity, and faster reaction kinetics make 0.2CuO/0.8CeO_2_ a promising material for fuel cell applications, where efficient proton conductivity and robust ORR are essential under H_2_-air conditions.


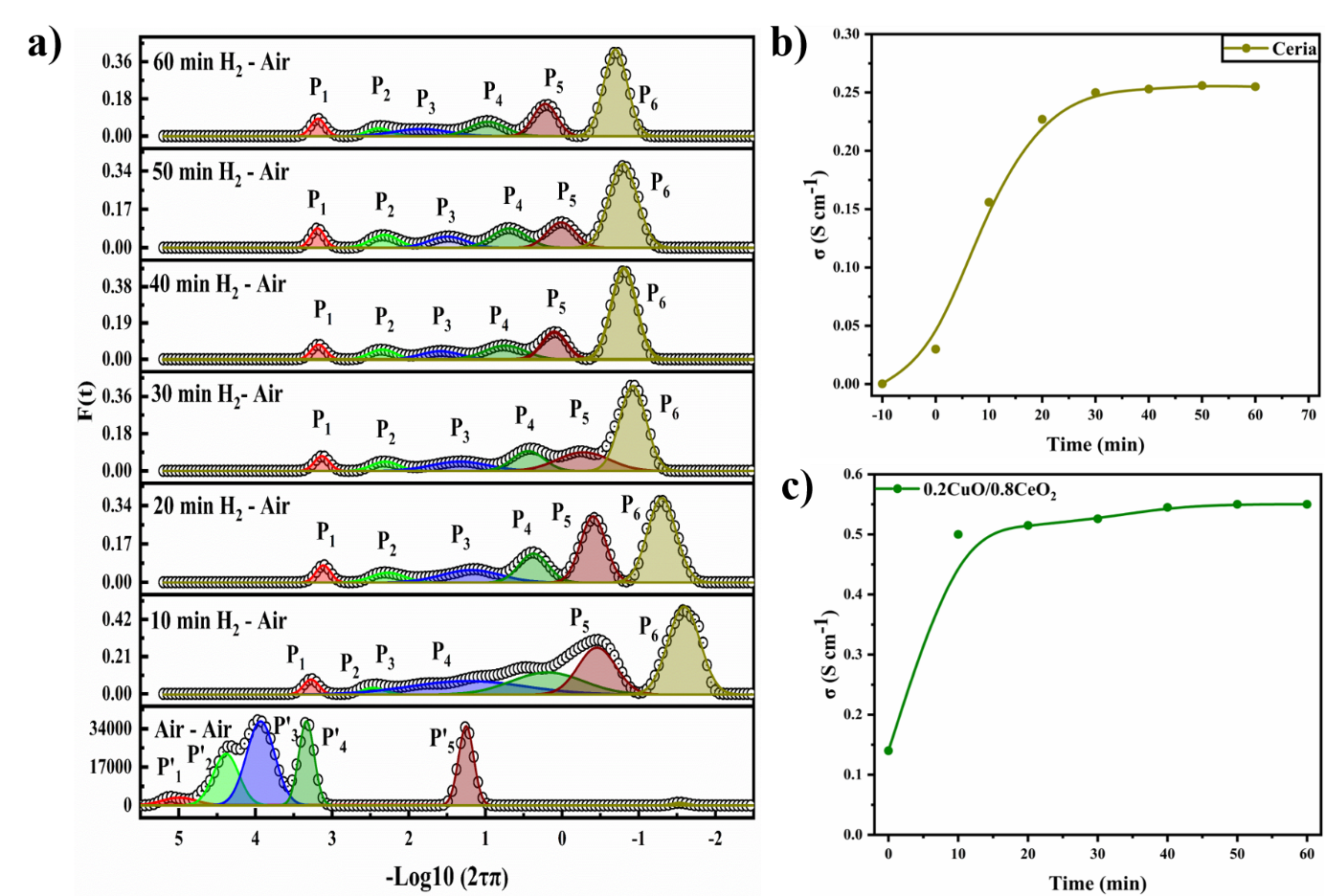


**Figure S3:** (a) The DRT results for CeO_2_ cell at 500 ℃ representing sub-processes involved to demonstrate effect of electrochemical proton injection and grain boundary conduction from air/air to H_2_/air atmosphere till 60 minutes. (b) Ionic conductivity plot of CeO_2_ as a function of time obtained from EIS. (c) For 0.2CuO/0.8CeO_2_.

Figure S4a-b provides a detailed electrochemical investigation for five layered based fuel cells of 0.2CuO/(0.8)CeO_2_ composition. Without BZY layer, cell provided maximum of 625 mW cm^-2^ power output with 1.04 V of OCV at 500 °C. While after applying the BZY layer it provides maximum of 515 mW cm^-2^ power output with 1.03 V which is approximately 82 % of without BZY layer, hinting toward protonic conduction nature of membrane. Moreover, it provided stable OCV of above 1.0 V till 350 °C, which is due to BZY layer electronic conduction suppression making the stable OCV and providing power output till 300 °C while no significant changes occur in EIS plot. It shows that the presence of several interfaces can also improve the electrical properties.


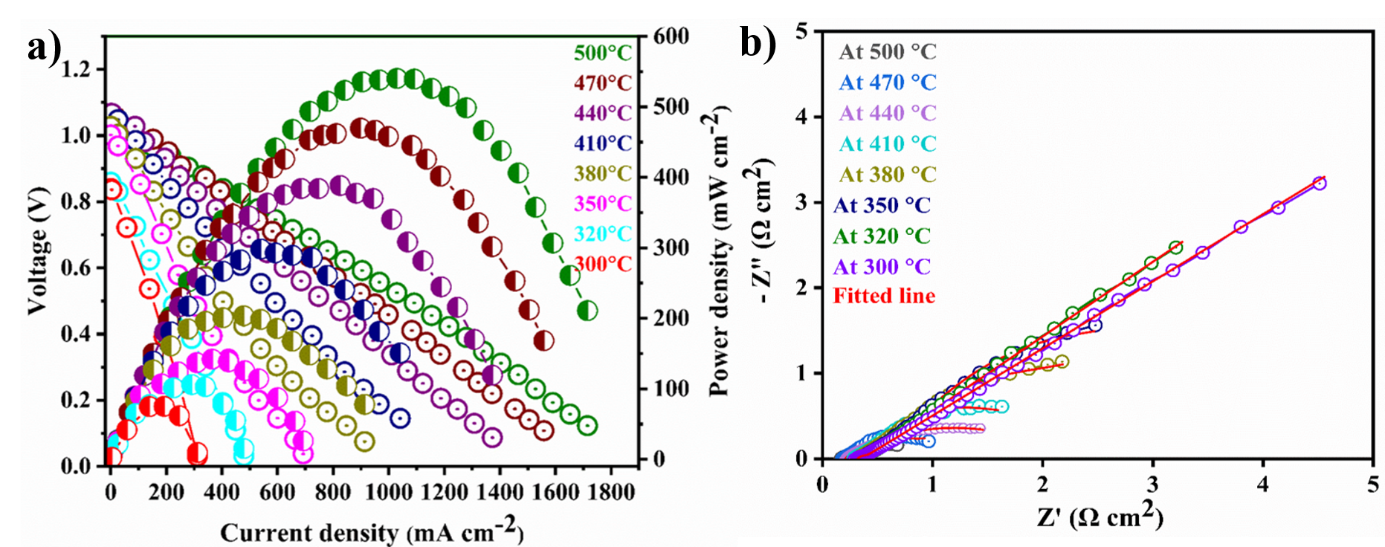


**Figure S4.** (a-b) Fuel cell performance in terms of power output and electrochemical impedance spectroscopy of the fuel cell device with five layers Ni-NCAL/BZY/ 0.2CuO/0.8CeO_2_/BZY/NCAL-Ni at different operational temperatures, respectively.


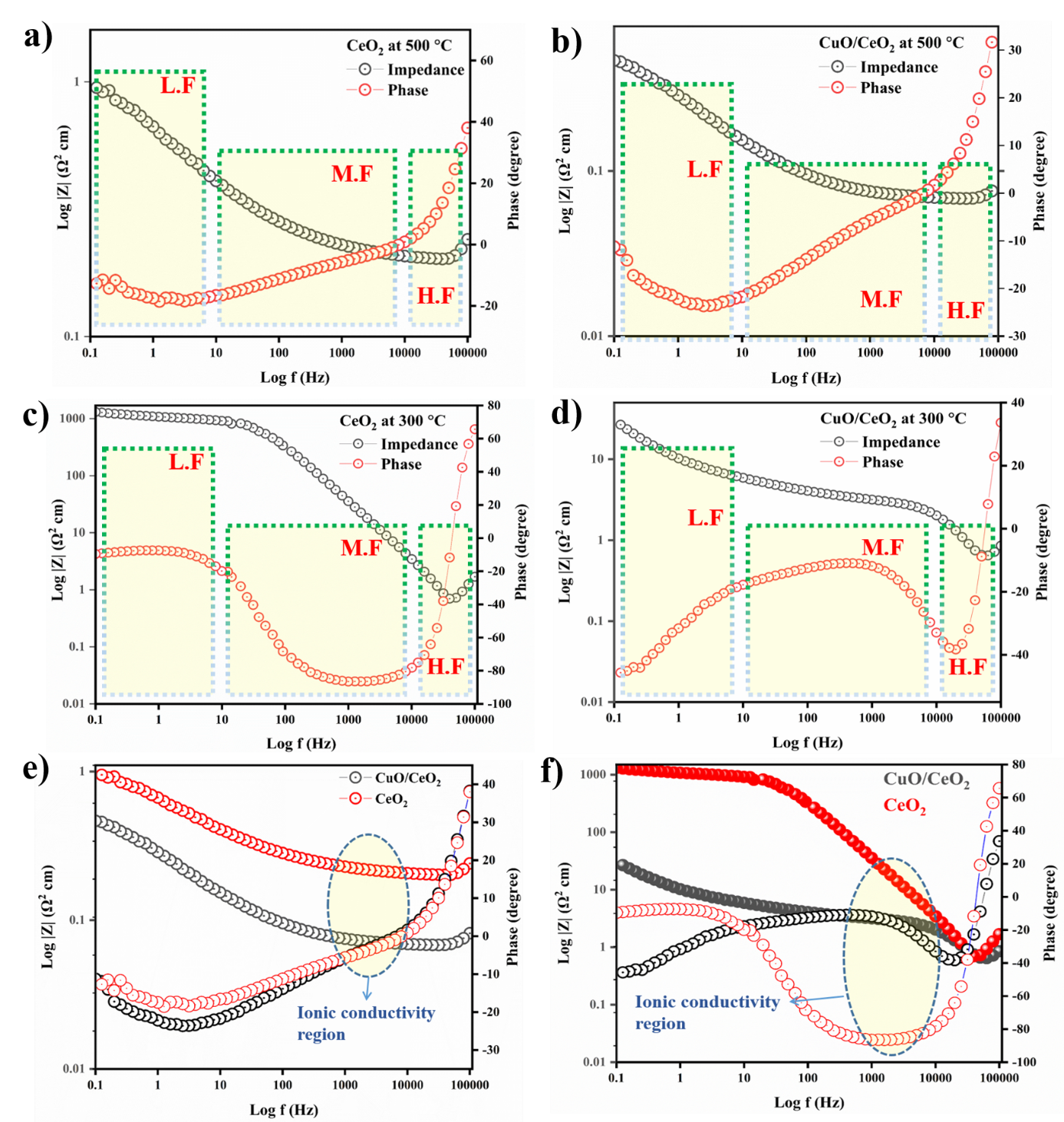


**Figure S5:** Bode plot for CeO_2_ and 0.2CuO/0.8CeO_2_, respectively (a-b) at 500 °C. (c-d) at 300 °C. (e) collective Bode plot analysis at 500 °C. (f) collective Bode plot analysis at 300 °C.


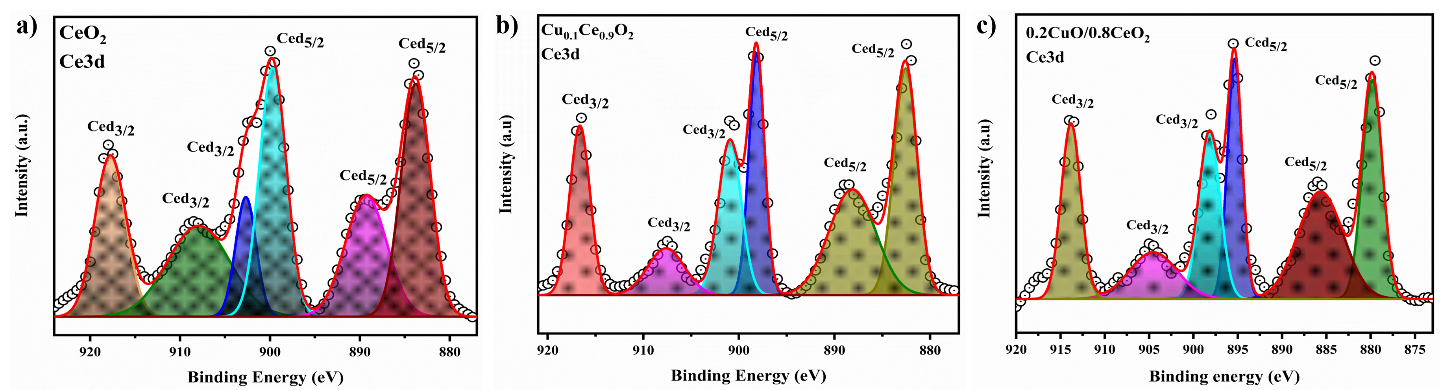


**Figure S6:** Ce-3d spectra for (a) CeO_2_, (b) Cu_0.1_Ce_0.9_O_2_, and (c) 0.2CuO/0.8CeO_2_.


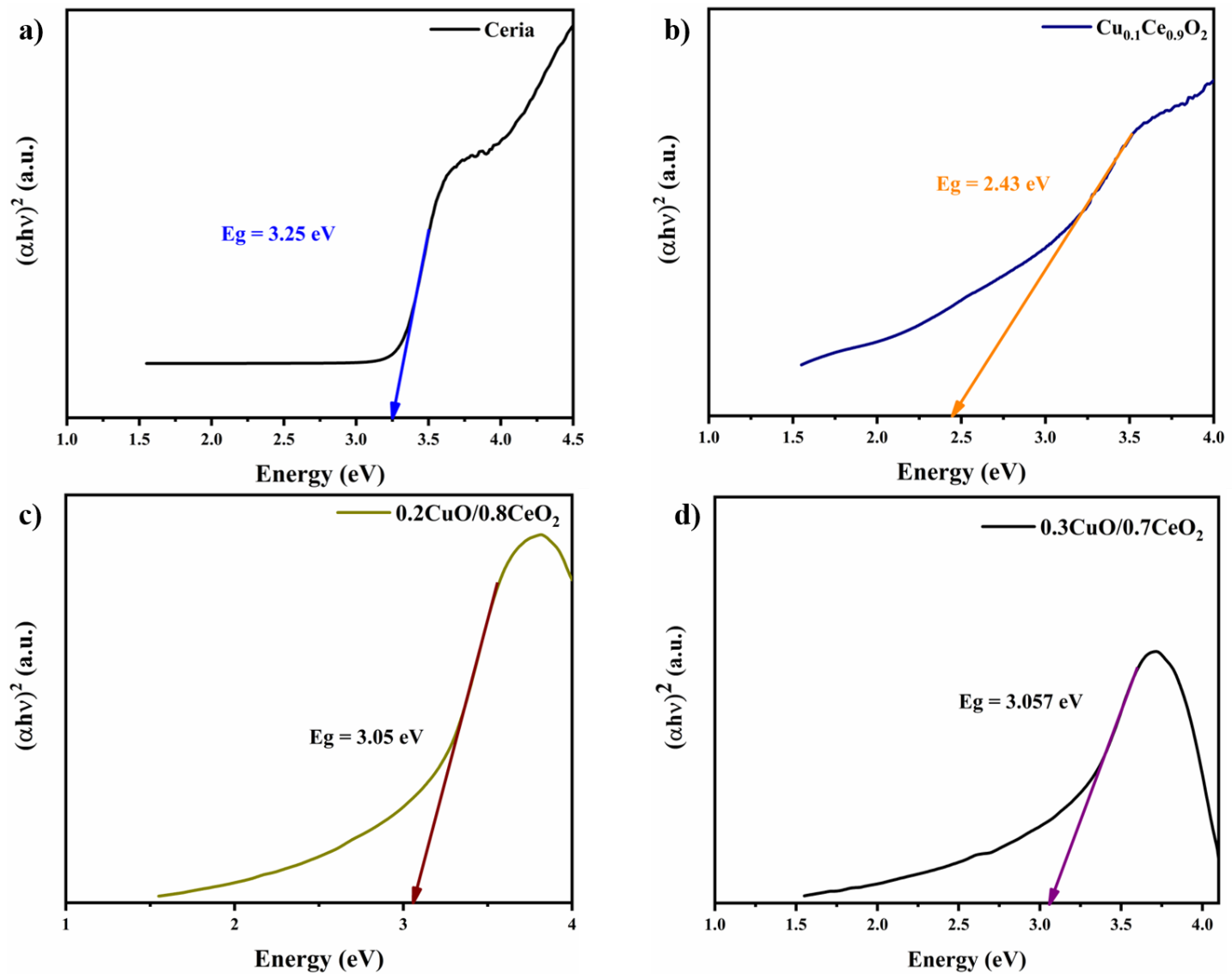


**Figure S7:** Band gap from the absorbance of UV-Vis graphs for (a). Ceria (b). Cu_0.1_Ce_0.9_O_2_ (c). 0.2CuO/0.8CeO_2_ (d). 0.3CuO/0.7CeO_2_.

The stabilization of the band energy at higher CuO-concentrations can be attributed to several factors. One prominent factor is the formation of Self-Nucleated CuO heterojunction. CuO introduces its own energy levels and electronic properties, offsetting the band gap reduction induced by ceria doping. Interfaces or phase boundaries between ceria and CuO create localized electronic effects, further influencing the band structure. Additionally, at higher CuO-concentrations, defect formation becomes more pronounced. These defects introduce energy levels within the band gap, counteracting the band gap reduction caused by Cu doping and leading to a more multifaceted electronic structure.


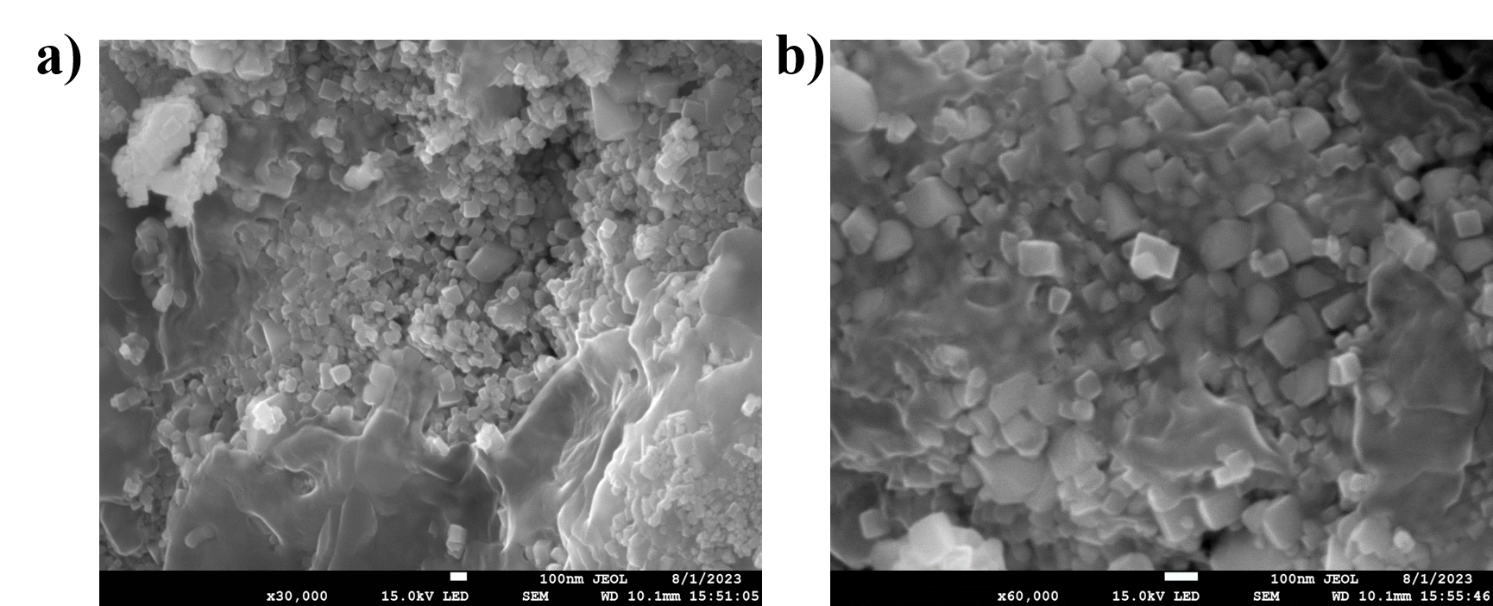


**Figure S8:** (a-b) SEM image of 0.2CuO/0.8CeO_2_.

**
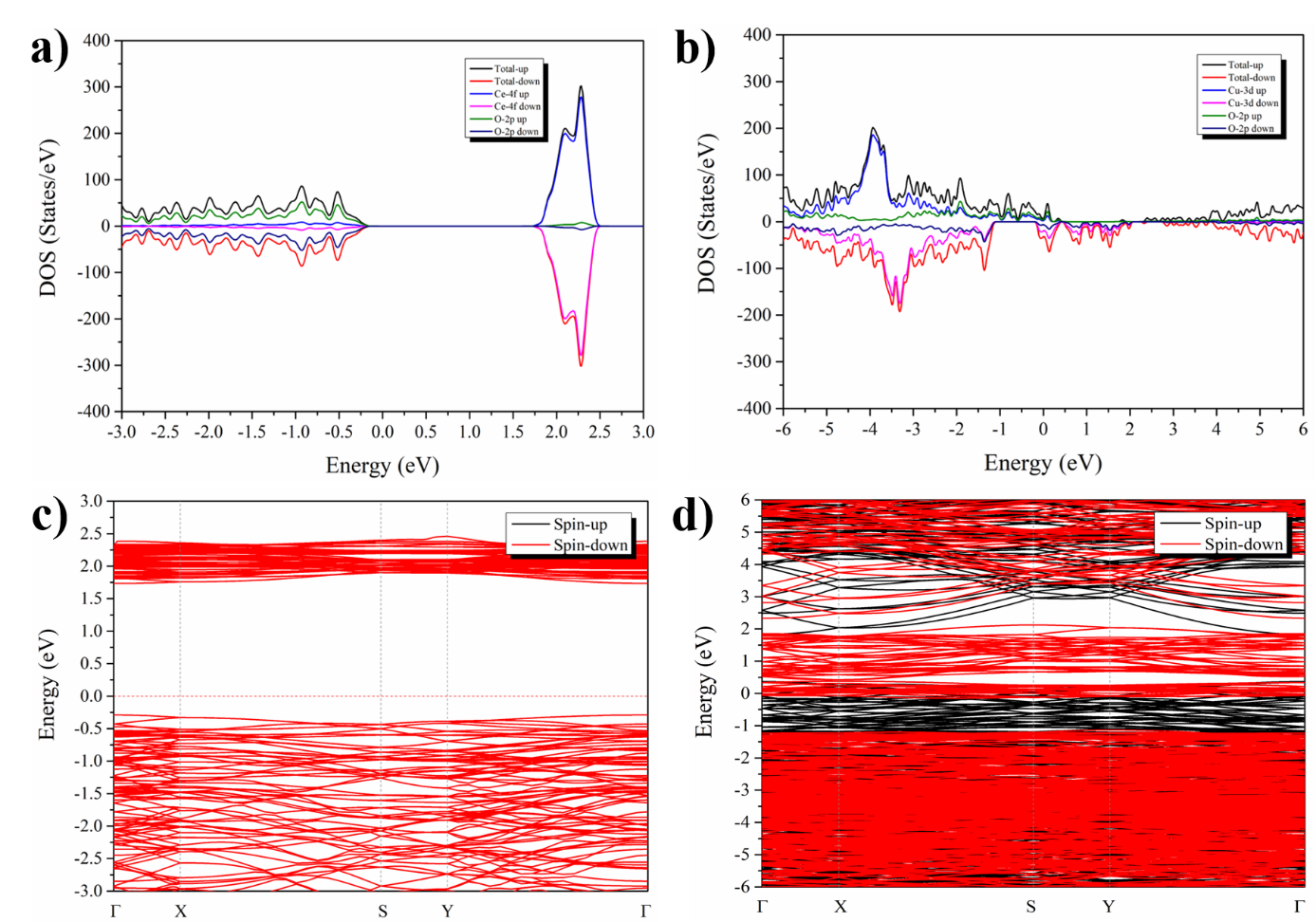
**

**Figure S9.** (a) Density of state of CeO_2_. (b) CuO; the d-band center was marked. (c) Energy band structure of CeO_2_. (d) CuO.

**Table S1** EIS Simulated data for CeO_2_ and Cu_0.1_Ce_0.9_O_2_ and Cu_0.2_Ce_0.8_O_2_; where the resistance is denoted by Ω cm^2^.

| **Temperature (°C)** | **R_0_** | **R_1_** | **R_T_ =R_0_ + R_1_** | **R_2_** |
| --- | --- | --- | --- | --- |
| **CeO_2_** |  |  |  |  |
| 500 | 0.19 | 0.10 | 0.29 | 0.62 |
| 470 | 0.20 | 0.11 | 0.33 | 1.10 |
| 440 | 0.22 | 0.16 | 0.38 | 1.92 |
| 410 | 0.49 | 0.34 | 0.83 | 5.45 |
| 380 | 0.75 | 12.09 | 12.85 | 43.16 |
| 350 | 1.02 | 182 | 183.02 | 176.68 |
| 320 | 1.72 | 950 | 951.72 | 184 |
| **Cu_0.1_Ce_0.9_O_2_** | **R_0_** | **R_1_** | **R_T_ =R_0_ + R_1_** | **R_2_** |
| 500 | 0.094 | 0.067 | 0.16 | 0.41 |
| 470 | 0.10 | 0.07 | 0.17 | 0.55 |
| 440 | 0.11 | 0.09 | 0.20 | 1.01 |
| 410 | 0.115 | 0.12 | 0.24 | 1.81 |
| 380 | 0.13 | 0.20 | 0.33 | 3.69 |
| 350 | 0.80 | 5.75 | 6.55 | 10.06 |
| 320 | 0.90 | 35.95 | 36.85 | 22.85 |
| 300 | 0.91 | 70 | 70.91 | 41.10 |
| **0.2CuO/(0.8)CeO_2_** | **R_0_** | **R_1_** | **R_T_ =R_0_ + R_1_** | **R_2_** |
| 500 | 0.075 | 0.034 | 0.11 | 0.31 |
| 470 | 0.082 | 0.037 | 0.12 | 0.53 |
| 440 | 0.093 | 0.053 | 0.15 | 0.69 |
| 410 | 0.11 | 0.08 | 0.19 | 1.78 |
| 380 | 0.12 | 0.10 | 0.22 | 4.10 |
| 350 | 0.15 | 0.14 | 0.29 | 6.47 |
| 320 | 0.63 | 0.65 | 1.28 | 16.7 |
| 300 | 0.82 | 1.92 | 2.74 | 19.26 |

**Table S2.** Area of peaks in Air – Air to H_2_- Air environment with different times for CeO_2_ composition.

| **Peak #** | **60** | **50** | **40** | **30** | **20** | **10** | **Air** |
| --- | --- | --- | --- | --- | --- | --- | --- |
| **P_1_** | 0.02031 | 0.01989 | 0.01989 | 0.01799 | 0.01841 | 0.02152 | 1996.33 |
| **P_2_** | 0.01515 | 0.02653 | 0.02252 | 0.0203 | 0.02379 | 0.01418 | 9655.50 |
| **P_3_** | 0.05037 | 0.03043 | 0.02819 | 0.04278 | 0.05371 | 0.14396 | 17338.9 |
| **P_4_** | 0.04139 | 0.04858 | 0.04867 | 0.0502 | 0.06111 | 0.14698 | 9463.42 |
| **P_5_** | 0.06097 | 0.05251 | 0.05723 | 0.07779 | 0.12157 | 0.16289 | 8798.29 |
| **P_6_** | 0.16385 | 0.17458 | 0.19623 | 0.17932 | 0.17547 | 0.26128 | 315.62 |

| **Peak #** | **Air** | **10** | **20** | **30** | **40** | **50** | **60** |
| --- | --- | --- | --- | --- | --- | --- | --- |
| **P_1_** | 0.03079 | 0.01549 | 0.01373 | 0.01391 | 0.01445 | 0.01337 | 0.01677 |
| **P_2_** | 0.06665 | 0.01244 | 0.00677 | 0.00875 | 0.01022 | 0.00796 | 0.01132 |
| **P_3_** | 0.56277 | 0.04815 | 0.06003 | 0.05462 | 0.05117 | 0.06172 | 0.0467 |
| **P_4_** | 0.48285 | 0.06123 | 0.00378 | 0.02936 | 0.02053 | 0.03402 | 0.03776 |
| **P_5_** | 1.99686 | 0.0762 | 0.09232 | 0.10815 | 0.09914 | 0.09724 | 0.07544 |
| **P_6_** |  | 0.07183 | 0.12353 | 0.0465 | 0.08472 | 0.06559 | 0.11436 |

**Table S3.** Area of peaks in Air – Air to H_2_- Air environment with different times for 0.2CuO/0.8CeO_2_ composition.

**Table S4.** Frequency of all peaks in Air – Air to H_2_- Air environment with different times for CeO_2_ composition.

|  | **Air** |  | **10** |  | **20** |  | **30** |  | **40** |  | **50** |  | **60** |  |
| --- | --- | --- | --- | --- | --- | --- | --- | --- | --- | --- | --- | --- | --- | --- |
| **Peaks** | **Start** | **End** | **Start** | **End** | **Start** | **End** | **Start** | **End** | **Start** | **End** | **Start** | **End** | **Start** | **End** |
| **P_6_** | 0.11424 | 0.28 | 0.05 | 0.61 | 0.08 | 0.9 | 0.23 | 2.3 | 0.33 | 2.94 | 0.2 | 2.9 | 0.55 | 4.5 |
| **P_5_** | 54.65 | 235.67 | 0.61 | 11.8 | 0.93 | 6.47 | 2.31 | 7.08 | 2.94 | 19.2 | 2.9 | 14.6 | 5.0 | 31.2 |
| **P_4_** | 6685.06 | 23723.37 | 11.85 | 433.62 | 6.47 | 42.22 | 7.08 | 53.7 | 19.2 | 104.6 | 14.6 | 84.6 | 31.2 | 222.9 |
| **P_3_** | 23723.37 | 105676.3 | 433.62 | 896.15 | 42.22 | 474.80 | 53.79 | 504.4 | 104.6 | 586.7 | 84.6 | 519.90 | 222.9 | 682.5 |
| **P_2_** | 105676.3 | 470737.3 | 896.15 | 4191.13 | 474.8 | 3192.31 | 504.41 | 3290.34 | 586.7 | 3391.3 | 519.9 | 3391.38 | 682.5 | 394.1 |
| **P_1_** |  |  | 5845.62 | 22801.52 | 4191.13 | 16348 | 4452.50 | 17367.5 | 4730.1 | 17900.85 | 4730.17 | 18450.57 | 5179.474 | 19601.19 |

**Table S5.** Frequency of all peaks in Air – Air to H_2_- Air environment with different times for 0.2CuO/0.8CeO_2_ composition.

|  | **Air** |  | **10** |  | **20** |  | **30** |  | **40** |  | **50** |  | **60** |  |
| --- | --- | --- | --- | --- | --- | --- | --- | --- | --- | --- | --- | --- | --- | --- |
| **Peaks** | **Start** | **End** | **Start** | **End** | **Start** | **End** | **Start** | **End** | **Start** | **End** | **Start** | **End** | **Start** | **End** |
| **P_6_** | 0.05 | 1.5 | 0.05 | 3.81 | 0.05 | 2.75 | 0.05 | 0.68166 | 0.0524 | 1.00652 | 0.05413 | 6.62025 | 0.0725 | 2.93944 |
| **P_5_** | 1.5 | 26.75 | 3.81 | 46.46616 | 2.75459 | 21.31238 | 0.68166 | 13.97253 | 1.00652 | 16.43603 | 6.62025 | 86.12315 | 2.93944 | 27.63547 |
| **P_4_** | 26.7 | 66.41 | 46.4 | 530.8414 | 21.31238 | 268.3941 | 13.97253 | 86.12315 | 16.43603 | 206.9846 | 86.12315 | 604.4805 | 27.63547 | 305.6261 |
| **P_3_** | 66.4 | 3606.80 | 530.84 | 1195.567 | 268.3941 | 1049.921 | 86.12315 | 1120.38 | 206.9846 | 1195.567 | 604.4805 | 983.8927 | 305.6261 | 1120.38 |
| **P_2_** | 3606.8 | 14575.07 | 1195.56 | 10881.06 | 1049.921 | 10533.36 | 1120.38 | 13222.01 | 1195.567 | 14109.33 | 983.8927 | 10881.06 | 1120.38 | 11240.24 |
| **P_1_** | 1.46 | 73931.47 | 11994.56 | 76371.91 | 10533.36 | 73931.47 | 13222.01 | 92802.52 | 14109.33 | 95865.97 | 10881.06 | 73931.47 | 11240.24 | 76371.91 |

**Table S6: Binding energy for each composition against lattice oxygen, defects and surface absorbed species.**

| **Composition** | **B.E for O_L_** | **B.E for O_v_** | **B.E for O-H peak** |
| --- | --- | --- | --- |
| Ceria | 531.2 | 534.1 | 535.9 |
| Cu_0.1_Ce_0.9_O_2_ | 528.9 | 531.5 | 534.1 |
| 0.2CuO/0.8CeO_2_ | 526.5 | 529 | 532.5 |
